# Shared and Unique Genetic Links between Neuroticism and Gastrointestinal Tract Diseases

**DOI:** 10.1155/2024/5515448

**Published:** 2024-06-21

**Authors:** Ye Tian, Jing Zi, Yifan Hu, Yaxian Zeng, Haoqi Li, Hang Luo, Jingyuan Xiong

**Affiliations:** ^1^Department of Occupational and Environmental Health, Healthy Food Evaluation Research Center, West China School of Public Health and West China Fourth Hospital, Sichuan University, Chengdu, China; ^2^Food Safety Monitoring and Risk Assessment Key Laboratory of Sichuan Province, Chengdu 610041, China

## Abstract

**Objective:**

Association between neuroticism and gastrointestinal tract (GIT) diseases may not be attributable to the genetic overlaps between neuroticism and psychiatric disorders. We aim to explore the genetic links and mechanisms of neuroticism and GIT diseases.

**Materials and Methods:**

We obtained European genome-wide association data of neuroticism (*n* = 390,278) or subclusters (depressed, *n* = 357,957; worry, *n* = 348,219) and six GIT diseases: gastroesophageal reflux disease (GERD, *n* = 456,327), inflammatory bowel disease (IBD, *n* = 456,327), peptic ulcer disease (PUD, *n* = 456,327), irritable bowel syndrome (IBS, *n* = 486,601), Crohn's disease (CD, *n* = 20,883), and ulcerative colitis (UC, *n* = 21,895). We performed genetic correlation analysis (high-definition likelihood method and cross-trait linkage disequilibrium score regression), pairwise pleiotropic analysis, single nucleic acid polymorphism annotation, Bayesian colocalization, gene-level analysis, transcriptome-wide association analysis, and gene set enrichment analysis.

**Results:**

Neuroticism and its subclusters are associated with most GIT diseases (15 of 18 trait-pairs). GERD and PUD were highly correlated with depressed affect. We identified pleiotropic loci 11q23.2 (mapped gene: *NCAM1*/*DRD2*) and 18q12.2 (mapped gene: *CELF4*) in neuroticism and IBS/GERD, supporting the genetic overlap between neuroticism and depression. We found that 16q12.1 (mapped gene: *NKD1*/*ZNF423*/*NOD2*) and 2q37.1 (mapped gene: *ATG16L1*/*SP140*) are only highlighted in depressed/neuroticism CD, revealing pleiotropic loci with dissimilarities between neuroticism and different GIT diseases. MR analysis suggested that genetic liability to neuroticism is associated with increased risks of IBS, PUD, and GERD.

**Conclusion:**

Our findings document the genetic links between neuroticism and six GIT diseases, highlighting the genetic overlaps and heterogeneity between neuroticism and psychiatric disorders in the context of gastrointestinal disorders. Both the shared and unique pleiotropic loci identified between neuroticism and different GIT diseases could facilitate mechanistic understandings and may stimulate further translational implications.

## 1. Introduction

Neuroticism, an important personality trait with public health significance, is considered as the tendency to experience negative emotions that, to some extent, predict less healthy life [1]. Genetic factors play a critical role in neuroticism with heritability estimates ranging from 40% to 50% [2]. While one study showed that neuroticism is strongly associated with depressive and anxiety symptoms, significant genetic heterogeneity exists between the two, and the genetic overlaps may be attributable to a major subcluster of neuroticism (depressed affect) [3, 4]. In addition, another study showed that neuroticism impacts physical health even when depression is under control [5]. Therefore, even previous evidence from genome-wide association studies (GWAS) reported shared genetic variants between gastrointestinal tract (GIT) diseases and psychiatric disorders [6]; it is not sufficient to fully understand the genetic links between neuroticism and GIT diseases including gastroesophageal reflux disease (GERD), peptic ulcer disease (PUD), inflammatory bowel disease (IBD), irritable bowel syndrome (IBS), Crohn's disease (CD), and ulcerative colitis (UC).

## 2. Materials and Methods

### 2.1. Data Sources

To ensure that the data samples were of the same ethnic origin, we obtained the GWAS summary data from publicly available data of European origin. The GWAS data for neuroticism and its subclusters (depressed affect and worry) were obtained from the same large meta-GWAS and were based on 449,484 individuals of European ancestry [7]. The number of samples included for each trait was reduced due to unpublished data from the 23andMe consortium (neuroticism, *n* = 390,278; depressed, *n* = 357,957; and worry, *n* = 348,219). For GIT diseases, the GWAS for GERD, IBD, and PUD were derived from the same GWAS dataset, based on 456,327 individuals from the UK Biobank [8]. The IBS GWAS was derived from another large GWAS meta-analysis that included a sample of 486,601 (case, *n* = 53,400; control, *n* = 433,201) [9]. We have included two major forms of the disease in IBD, including UC (case, *n* = 6,968; control, *n* = 14,927) and CD (case, *n* = 5,956; control, *n* = 14,927) [10]. Details of the above datasets were shown in *Supplementary table 2*.

### 2.2. Study Design and Quality Control

**Figure 1:**
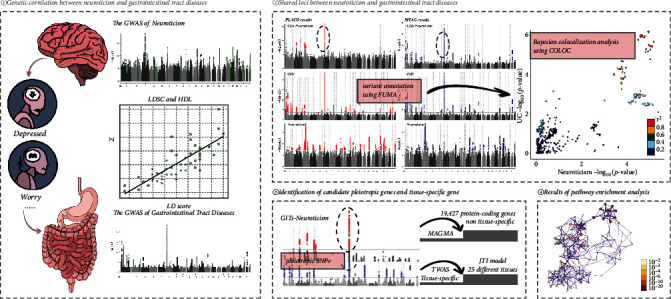
Study design. We conducted a genome-wide pleiotropic analysis of four dimensions of neuroticism and six gastrointestinal diseases. HDL, high-definition likelihood; LDSC, linkage disequilibrium score regression; GWAS, genome-wide association study; MTAG, multitrait analysis of GWAS; PLACO, pleiotropic analysis under the composite null hypothesis; MAGMA, multimarker analysis of genomic annotation; TWAS, transcriptome-wide association study; JTI, joint-tissue imputation; GIT diseases, gastrointestinal tract diseases.

The study overview is shown in Figure 1. Inclusion criteria and data quality control were reported in the original publication [7, 8, 9, 10]. We performed additional quality controls on the above GWAS data: (1) removal of single nucleic acid polymorphisms (SNPs) without rsID; (2) exclusion of SNPs in the major histocompatibility complex region (MHC, chr 6 : 25–35 Mb) due to its complex LD structure; and (3) uniform alignment with the hg19 human reference genome.

### 2.3. Genetic Correlation Analysis

High-definition likelihood (HDL) method and cross-trait linkage disequilibrium score (LDSC) regression analyses were conducted to evaluate the genetic correlation (*r*_g_) for 24 pairwise traits between neuroticism and six GIT diseases [11, 12, 13]. LDSC intercept could indicate probable sample overlap between two sets of GWAS. HDL extends the LDSC by modeling the relation between covariances among *Z* statistics for pairs of traits across multiple SNPs and a full matrix of cross-SNP LD scores. Compared to LDSC, HDL utilizes LD information from the entire genome, improving the accuracy of the results. So, we use HDL as the main result. We did not restrict the intercept of the LDSC to consider the overlap of the samples.

We performed LDSC using known LD scores from 1,000 Genomes Project European pedigree reference data and well-imputed HapMap3 variants. We conducted HDL by LD reference computed from 335,265 genomic British individuals in UKB.

### 2.4. Pairwise Pleiotropic Analysis

To identify SNPs related to joint phenotypes of pairwise traits, we used multitrait analysis of GWAS (MTAG) and the recently developed pleiotropic analysis under the composite null hypothesis (PLACO) [14, 15]. By taking GWAS summary data for multiple traits as input and applying generalized inverse variance weighted meta-analysis to improve statistical power, MTAG detects novel genetic associations for each trait. To investigate the assumptions, the upper bound for the false discovery rate was calculated. Since summary data for each trait obtained using MTAG can substitute the summary GWAS data for individual traits, MTAG was used for subsequent analysis. The genome-wide significance for MTAG results of neuroticism-GIT was set at *p*_MTAG_ < 5 × 10^−8^.

In addition, since MTAG is subject to the homogeneous assumption leading to overall inflation, which may affect the credibility of the results, we validated the results using PLACO, which detects multidirectional associations by considering a compound null hypothesis, where the null hypothesis H_0_ is a compound of the global null hypothesis (*β*_trait1_ = *β*_trait2_ = 0), with subzero hypotheses (*β*_trait1_ = 0, *β*_trait2_ ≠ 0) and (*β*_trait1_ ≠ 0, *β*_trait2_ = 0). The test statistic for PLACO is *T*_PLACO_ = *Z*_trait1_*Z*_trait2_. Of these, *β*_trait1_ and *β*_trait2_ represent the effect sizes for neuroticism and GIT disorders, respectively, and correspondingly, *Z*_trait1_ and *Z*_trait2_ as the observed *Z*-scores of an SNP from corresponding GWAS summary data, respectively. Given the possible existence of sample overlap, we decorrelated the *Z*-scores using the correlation matrix obtained from GWAS summary statistics. The genome-wide significance level for PLACO results of neuroticism-GIT was set at *p*_PLACO_ < 5 × 10^−8^.

### 2.5. SNP-Level Annotation

To obtain significant pleiotropic loci and lead SNPs, we annotated the MTAG summary data and the results of PLACO using the online platform functional mapping and annotation (FUMA) [16]. Lead SNPs, a subset of significant SNPs, are defined if they are independent of each other at *r*^2^ < 0.1. We performed FUMA annotation using the default settings and 1,000 Genomes Project Phase 3 of the European ancestry as a reference panel. We compared the pleiotropic loci derived from the MTAG and PLACO results and further identified them as FUMA-annotated pleiotropic loci. We conducted additional functional evaluations by annotate variation (ANNOVAR) categories, combined annotation-dependent depletion (CADD) scores, and RegulomeDB (RDB) scores [17, 18, 19].

### 2.6. Bayesian Colocalization

To further determine whether there was causal variation in the pleiotropic loci for each pair of traits, we performed Bayesian colocalization of the FUMA-identified pleiotropic loci using the R package coloc [20]. Bayesian colocalization analysis relied on a single dependent variable hypothesis and provided posterior probabilities (PP) for the five hypotheses at each pleiotropic locus. These five PP were H0 (neither trait has a genetic association), H1 and H2 (association to one trait only), H3 (both traits are associated but with different causal variants), and H4 (both traits are associated and share a single causal variant) [20]. We calculated posterior probabilities for H4 (PP.H4) and H3 (PP.H3) and declared genomic loci with a PP.H4 greater than 0.75 as a colocalized locus with the potential of being a shared causal variant between two traits.

### 2.7. Gene-Level Analysis

Compared to SNP-level analysis, gene-based association analysis provides greater power to identify genetic risk variants. Because gene-based analysis aggregates the effects of multiple SNPs and genes are more closely related to biology than SNPs, it can help us better understand the underlying biological mechanisms underpinning cross-trait traits.

We first performed gene-level multimarker analysis of genomic annotation (MAGMA) of pleiotropic SNPs based on PLACO results and MTAG results to identify candidate pleiotropic genes [21]. MAGMA Gene IDs and locations of 19,427 protein-coding genes based on NCBI build 37.3. Based on genetic association analysis, we were able to identify significant pleiotropic genes for each pair of traits. The significance gene was declared at both the locus-specific Bonferroni-corrected *p* < 0.05/N (N: total number of genes analyzed for each pair of traits) for MAGMA analysis on PLACO results and MTAG results.

In addition, to further explore the tissue-specific expression of genes, we performed analysis using the transcriptome-wide association study (TWAS), which allows the integration of GWAS and expression quantitative trait loci (eQTL) data to identify tissue-specific genes [22]. We included 25 specific tissues (13 brain tissues, seven gastrointestinal tissues, whole blood, and four additional tissues including pituitary, adrenal gland, liver, and EBV-transformed lymphocytes) as a reference panel (*Supplementary table 2*), using the results of MTAG and PLACO and the S-PrediXcan program in combination with the joint-tissue imputation (JTI) model for a two-stage TWAS analysis [23, 24]. Significance for single tissue test was established by Bonferroni correction, equivalent to 0.05 divided by the number of genes tested in each tissue.

### 2.8. Gene Set Enrichment Analysis

To better understand the underlying biological mechanisms of neuroticism and GIT diseases or their comorbidity, we performed the gene set enrichment analysis (GSEA) on the results of MAGMA and TWAS using the Metascape online tool, using gene sets derived from Gene Ontology (GO) and Kyoto Encyclopedia of Genes and Genomes (KEGG) pathway database [25, 26]. To integrate the overall effects of the neurotic and gastrointestinal systems, all results from MAGMA and TWAS were integrated separately and analyzed using Metascape.

### 2.9. Mendelian Randomization

Based on the genetic correlation results, we further explored the causal association between neuroticism and GIT diseases at the genetic level using two-sample MR. Considering the overlap in the sample populations used for the GWAS on neuroticism and GIT diseases, employing a two-sample MR approach may inaccurately estimate the effect size. Consequently, we utilized GWAS dataset (depression or dysthymia, case, *n* = 54,733; control, *n* = 242,809) from FinnGen as the exposure to approximate the causal effect between neuroticism and GIT diseases to mitigate the potential bias introduced by sample overlap [27]. In the selection of significant SNPs for exposures, we employed a rigorous significance threshold (*p* < 5 × 10^−8^). Utilizing the 1,000 Genomes Project Phase 3 (European) as the reference panel, along with these identified significant SNPs, we performed LD clumping to identify instrumental variables (*r*^2^ < 0.001 within 10,000 kb). Furthermore, to mitigate the impact of confounding factors, we utilized the PheLiGe website to examine the correlations between the instrumental variables and other phenotypes [28].

In our MR framework, the inverse variance-weighted (IVW) method served as the primary analysis [29]. To enhance the reliability of IVW estimates in the context of horizontal pleiotropy, we employed MR-Egger regression and the weighted median method for sensitivity analysis [30, 31]. Furthermore, the MR-PRESSO was implemented to identify and adjust for potential outliers, thereby refining the evaluation of the causal effect [32]. To account for multiple comparisons, we used Bonferroni correction (corrected *p*: 0.05/6 = 8.3 × 10^−3^). We adopted the following criterion: a potential causal effect is suggested when the Bonferroni corrected *p* from the IVW analysis is less than 0.05. Given the conservative nature of the Bonferroni correction, we consider results with a corrected *p* greater than 0.05 and an uncorrected *p* less than 0.05 to indicate a potential causal association.

### 2.10. Data and Code Availability

The data and code availability is as follows: HDL (https://github.com/zhenin/HDL); LDSC (https://github.com/bulik/ldsc); FUMA (https://fuma.ctglab.nl/); PLACO (https://github.com/RayDebashree/PLACO); MTAG (https://github.com/JonJala/mtag); COLOC (https://github.com/chr1swallace/coloc); MetaXcan (https://github.com/hakyimlab/MetaXcan); and GWAS catalog (https://www.ebi.ac.uk/gwas/home).

## 3. Results

### 3.1. Genetic Correlation between Neuroticism and Gastrointestinal Tract Diseases

**Figure 2:**
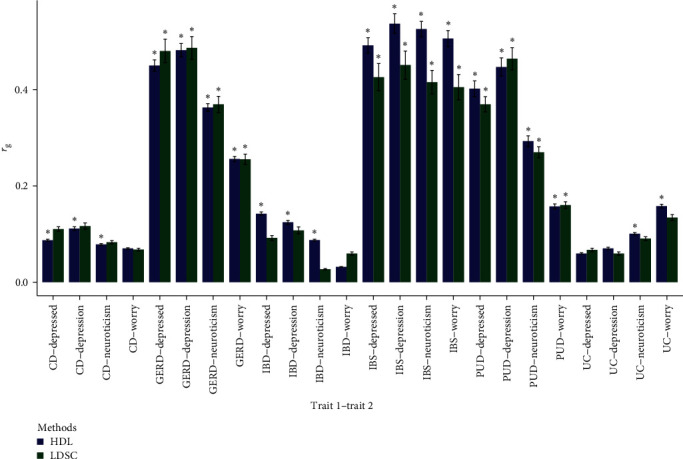
Eighteen pairs of traits with significant genetic correlations identified by HDL and LDSC methods. Error bars in black showed the 95% confidence intervals of genetic correlation estimates. *⁣*^*∗*^indicates less than the Bonferroni-corrected significance threshold (2.78 × 10^−3^). HDL indicates high-definition likelihood; LDSC, linkage disequilibrium score regression; IBD, inflammatory bowel disease; IBS, irritable bowel syndrome; PUD, peptic ulcer disease; GERD, gastroesophageal reflux disease; CD, Crohn's disease; UC, ulcerative colitis.

We found significant genetic correlations in nine out of 18 trait pairs using LDSC and 15 out of 18 trait pairs using the more precise method HDL (*Supplementary table 2*). Notably, the results showed high genetic correlations of GERD, IBS, and PUD with neuroticism and subclusters (Figure 2). Among them, GERD, IBD, and PUD are mainly affected by depressive mood. UC is mainly affected by worry. We included the above 15 pairs of traits in the subsequent analysis.

### 3.2. Shared Loci between Neuroticism and Gastrointestinal Tract Diseases

We first performed a meta-analysis of GWAS of GIT and GWAS of neuroticism using MTAG, and a total of 15 MTAG results were obtained. All mean *χ*^2^ increase from GIT of GWAS to GIT of MTAG (*Supplementary table 2*). However, considering that the maxFDR of some trait pairs is greater than 0.05, leading to possible inflation and false positive results (*Supplementary table 2*), we performed parallel analysis using PLACO analysis to further validate the results obtained from MTAG analysis. We used FUMA to identify 272 shared loci containing 96 unique chromosomal regions from the MTAG results of 15 pairs of traits (*Supplementary table 2* and *Supplementary figure 1*). We used FUMA to identify 132 shared loci containing 55 unique chromosomal regions from the PLACO results of 15 pairs of traits (*Supplementary table 2* and *Supplementary figure 1*). By comparison, we finally identified 77 independent genomic risk loci as pleiotropic loci, involving 33 unique chromosomal regions (*Supplementary table 2* and *Supplementary figure 1*). In addition, we found dissimilarities in the pleiotropic loci between neuroticism and GIT diseases. For example, 11q23.2 (mapped gene: *NCAM1*/*DRD2*) and 18q12.2 (mapped gene: *CELF4*) is widely highlighted between neuroticism-IBS and neuroticism-GERD, and we found that 16q12.1 (mapped gene: *NKD1*/*ZNF423*/*NOD2*) and 2q37.1 (mapped gene: *ATG16L1*/*SP140*) are only highlighted in neuroticism/depressed-CD.

We identified 44 lead SNPs by comparing the FUMA results of MTAG and PLACO, containing 31 unique lead SNPs (*Supplementary table 2*). Of these, rs11665070 (location: 18q12.2; mapped gene: *CELF4*; and CADD score: 11.24) located in an intergenic variant is widely expressed in several trait pairs.

Colocalization analysis further identified 22 potential pleiotropic loci from the 77 shared loci identified by FUMA (Table 1 and *Supplementary figure 1*). A total of 16 causal single nucleotide variants (SNVs) were similarly identified in FUMA (Table 1 and *Supplementary figure 1*). Notably, 11q23.2 (mapped gene: *NCAM1*) was identified as a pleiotropic locus in multiple trait pairs, mainly associated with GERD and IBS. In addition, 22q13.2 (mapped gene: *L3MBTL2*) and 14q24.3 (mapped gene: *YLPM1*/*DLST*) were identified as pleiotropic loci for neuroticism-IBS. 17q21.1 (mapped gene: *MED24*/*ZPBP2*) was identified as a pleiotropic locus for multiple trait pairs of neuroticism-UC. 2q33.1 was identified as a pleiotropic locus for neuroticism-IBS/UC.

### 3.3. Identification of Candidate Pleiotropic Genes and Tissue-Specific Gene

Based on the pleiotropic loci identified by PLACO and MTAG, we further identified 324 significant pleiotropic genes, including 150 unique genes, in 15 trait pairs using MAGMA (*Supplementary table 2*). *NCAM1* and *TNXB* were identified as pleiotropic genes in nine trait pairs. *H2BC15*, *MSH5*, *H2BC13*, *DRD2*, *H3C12*, and other pleiotropic genes were also identified in several trait pairs.

We further used the JTI model to identify 236 unique tissue-specific genes from 15 trait pairs and 25 related tissues. *C4A* was identified in greater numbers in multiple traits and tissues (25 tissues). *BTN3A2*, *FLOT1*, *ZSCAN9*, and *RABGAP1L-DT* are also expressed in several tissues but restricted to trait pairs of GERD and IBS with neuroticism. In addition, several genes are only expressed in a single GIT disease with neuroticism. For example, *PCCB* and *RERE-AS1* are only expressed in IBS and neuroticism. *ZSCAN26* and *PRSS16* are expressed only in GERD and neuroticism (*Supplementary table 2*). Tissue-specific expression of the genes is concentrated in whole blood, caudate basal ganglia, putamen basal ganglia, cerebellar hemispheres, and cerebellum; genes are less expressed in the substantia nigra of the brain and liver (*Supplementary figure 1*).

### 3.4. Pathway Enrichment Analysis

**Figure 3:**
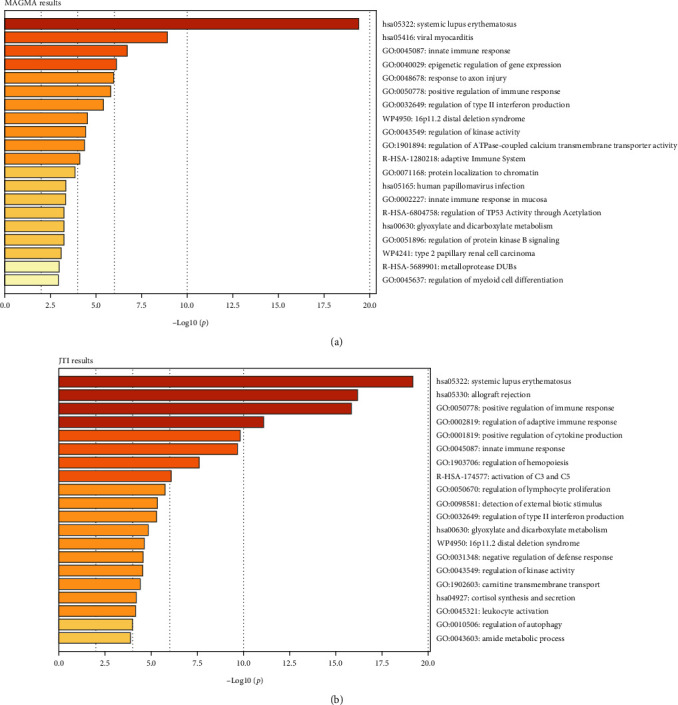
Enrichment analysis of MAGMA and JTI results: (a) and (b) bar graph of enriched terms across input gene lists, colored by *p* values.

We performed pathway enrichment analysis of all unique genes identified by MAGMA and TWAS using Metascape, respectively (Figure 3). Interestingly, we noticed that these pathways are closely linked to altered immune signaling. For example, both enrichment results suggested an association with innate immune response (GO:0045087), and positive regulation of immune response (GO:0050778).

### 3.5. Casual Associations between Neuroticism and GIT Diseases

We identified 29 significant SNPs from GWAS (depression or dysthymia). Based on our investigation using the PheLiGe website, we identified five SNPs that may be associated with confounding factors, including BMI, alcohol consumption, smoking, and celiac disease (*Supplementary table 2*). Ultimately, a total of 24 SNPs were included as instrumental variables in the analysis (*Supplementary table 2*). In the primary IVW analysis, genetic liability to depression or dysthymia was significantly associated with an increased GERD (OR = 1.15; 95%CI, 1.05–1.25; *p* = 1.62 × 10^−3^) and IBS (OR = 1.28; 95%CI, 1.13–1.45; *p* = 8.67 × 10^−5^) risk (*Supplementary figure 1*). In addition, MR result indicated that liability to depression or dysthymia may be associated with an increased risk of PUD (OR = 1.16; 95%CI, 1.02–1.30, *p*=0.02) (*Supplementary figure 1*). However, the MR results did not identify an association between liability to depression or dysthymia and the risks of CD, IBD, and UC.

## 4. Discussion

Based on the pleiotropic SNP analysis of MTAG and PLACO, we identified 77 independent genomic risk loci as pleiotropic, involving 33 unique chromosomal regions. Colocalization analysis was further utilized to identify 22 potential causal sharing variants. For example, 11q23.2 (mapped gene: *NCAM1*/*DRD2*) has a wide distribution of pleiotropic variants between neuroticism and IBS as well as GERD. And in a previous study, it was shown that 11q23.2 was present in major depressive disorder with IBS and GERD, which partly explains the genetic overlap that exists between neuroticism and depression [6, 9]. *DRD2* (encoding D2 dopamine receptor), located in the same region, has been suggested to play an important role in the pathogenesis of anxiety and depression, and psychological stress drives the development of malignancy via *DRD2* signaling [33, 34]. In addition, *DRD2* is associated with the etiology of neuropsychiatric disorders as an important gene coding for the monoaminergic neurotransmission system [35]. Dopamine receptors are abundant in the gastrointestinal tract, and previous animal studies suggest that dopamine receptors promote pepsinogen secretion by inhibiting the release of growth inhibitory hormone from the gastric mucosa, while adverse gastrointestinal reactions in levodopa-treated patients suggest that excessive dopamine stimulation may inhibit intestinal peristalsis causing gastrointestinal-related symptoms [36, 37]. This implies that *DRD2* antagonists can alleviate such gastrointestinal symptoms to a certain extent. Interestingly, phenothiazines are widely used as *DRD2* antagonists in anxiety disorders, and *DRD2* inhibitors can have a therapeutic effect on tumor-bearing mice under pressure stimulation. This evidence indicates that *DRD2* antagonists can ameliorate adverse symptoms in response to psychological stimuli, further suggesting a role for *DRD2* in the prodromal symptoms of psychiatric disorders [34].

In addition to pleiotropic loci that are widely highlighted in different trait pairs, we also found pleiotropic loci that are uniquely highlighted in different trait pairs. For example, we reported for the first time that the pleiotropic loci 16q12.1 (mapped gene: *NKD1*/*ZNF423*/*NOD2*) and 2q37.1 (mapped gene: *ATG16L1*/*SP140*) are highlighted in CD and neuroticism. As an important protein involved in autophagy function, the nucleotide polymorphism of *ATG16L1* is related to CD [38]. Previous studies have suggested that zinc is essential for early and late autophagy, zinc deficiency exacerbates CD activity, and zinc can repair the gut via zinc finger protein A20 [39, 40]. In addition, autophagy can suppress inflammation by degrading inflammatory vesicles [39]. Of note, there is evidence of an association between low serum zinc levels and depression, while studies have shown a bidirectional link between depression as well as anxiety and the inflammatory response and course of IBD [41, 42]. Interestingly, the inflammatory response in CD is regulated by the *ATG16L1* polymorphism through modulation of TLR- and NLR-mediated signaling [43]. NLRs are important in the regulation of the gastrointestinal tract as innate immune pattern recognition receptors expressed in the gut and brain. Whereas *NOD2* on our identified pleiotropic locus 16q12.1, as a recognition receptor in the NLR family, has been shown that mice deficient in *NOD2* and *NOD1* exert signs of stress anxiety and depression in the context of hypothalamic–pituitary–adrenal axis hyperactivity [44]. Our study further provided evidence at the genetic level for a shared genetic basis for CD and neuroticism. In the colocalization analysis, we further identified 24 pleiotropic loci with potential causal potency. For example, 11q23.1 (mapped gene: *NCAM1*) is again shown to be widely highlighted in multiple trait pairs. In addition, we identified 22q13.2 (mapped gene: *L3MBTL2*) as a pleiotropic locus for IBS and neuroticism. This locus was previously reported to be linked with schizophrenia [45]. There is evidence that variants in *L3MBTL2* affect early embryonic development and that schizophrenia is associated with early neurodevelopmental disorders [46, 47]. This suggests that gastrointestinal system may be affected by early neurodevelopment.

The results of our enrichment pathway analysis show that the common biological pathways of neuroticism and GIT diseases mainly involve membrane vesicular transport as well as the immune system. For the membrane vesicular transport, the transmission of important neurotransmitters is involved, e.g., serotonin (5-hydroxytryptamine; 5-HT) as an important neurotransmitter whose role in the gut–brain axis has been emphasized. 5-HT can affect gastrointestinal inflammation, as well as the mechanics of gastrointestinal muscles, through different mechanisms [48, 49]. In addition, secondary bile acids and short-chain fatty acids produced by the gut microbiota stimulate the secretion of 5-HT by enterochromaffin cell (EC cell) [50]. Recent studies have shown that activation and inhibition of EC cells cause anxiety by disrupting gastrointestinal tract function [51].

Further MR analysis suggested that genetic liability to neuroticism is associated with increased risks of IBS, PUD, and GERD. It is important to note that, to avoid bias due to sample overlap between neuroticism and GIT diseases GWAS samples, we utilized GWAS data from FinnGen as a substitute for neuroticism phenotype GWAS data. Previous research suggests that emotional states may impact physical health, leading to an increased susceptibility to diseases [52]. A randomized controlled trial aimed to investigate the relationship between gastrointestinal symptoms and psychological factors in patients with IBS revealed that 44% of the IBS patients had psychiatric comorbidities [53]. From a neurobiological perspective, central nervous system pathways constitute the emotional motor system, which can be regarded as an extension of the limbic system to the gut, participating in the regulation of neuroendocrine pathways [54]. Furthermore, a previous genome-wide multitrait analysis also explored the extensive genetic sharing between IBS, neuroticism, depression, and anxiety, identifying 42 significant SNPs [55]. Compared to this prior study, our research includes a broader range of GIT diseases. For instance, we further emphasize that the locus 11q23.2 (mapped gene: *NCAM1*) is significantly expressed not only in IBS but also in GERD, and we highlight the potential importance of the *DRD2* gene at the same loci. In summary, our study further enriches the understanding of the complex genetic interplay between psychological traits and GIT diseases.

## 5. Conclusion

Our study suggests genetic links between neuroticism and GIT diseases, reveals both shared and unique pleiotropic loci, highlights potential roles of gut–brain axis in neuroticism, and supports future mechanistic and translational studies for neuroticism and different GIT diseases.

## Figures and Tables

**Table 1:** Twenty-two colocalization analysis performed on 77 shared loci.

Trait 1	Trait 2	Region	PLACO			MTAG			PP.H3	PP.H4	Best causal	SNV.PP.H4
			Top SNP	*p*	Nearest gene	Top SNP	*p*	Nearest gene				
CD	Depressed	3p21.31	rs1873625	1.74 × 10^−17^	*BSN*	rs13096760	2.63 × 10^−12^	*NICN1*	0.222	0.778	rs1873625*⁣*^*∗*^	0.139
CD	Depressed	4q24	rs13135092	5.26 × 10^−09^	*SLC39A8*	rs13135092	2.49 × 10^−09^	*SLC39A8*	0.001	0.817	rs13107325	0.971
GERD	Depressed	11q23.2	rs2186709	5.49 × 10^−12^	*NCAM1*	rs1940722	1.65 × 10^−11^	*NCAM1*	0.099	0.9	rs10502163	0.036
IBS	Depressed	11q23.2	rs10891490	9.20 × 10^−13^	*NCAM1*	rs10891490	3.47 × 10^−12^	*NCAM1*	0.177	0.823	rs10891490*⁣*^*∗*^	0.019
IBS	Depressed	14q24.3	rs10144845	2.80 × 10^−11^	*YLPM1*	rs10144845	2.03 × 10^−10^	*YLPM1*	0.046	0.809	rs10144845*⁣*^*∗*^	0.224
IBS	Depressed	22q13.2	rs136402	1.17 × 10^−08^	*L3MBTL2*	rs136402	1.45 × 10^−09^	*L3MBTL2*	0.114	0.862	rs136402*⁣*^*∗*^	0.074
CD	Neuroticism	6q27	rs444210	3.56 × 10^−12^	*RP1-167A14.2*	rs444210	7.21 × 10^−14^	*RP1-167A14.2*	0.186	0.793	rs444210*⁣*^*∗*^	0.079
GERD	Neuroticism	2p15	rs4671537	4.55 × 10^−10^	*VPS54*	rs66595136	1.80 × 10^−09^	*VPS54*	0.125	0.87	rs66595136*⁣*^*∗*^	0.107
IBS	Neuroticism	11q23.2	rs10891490	6.32 × 10^−15^	*NCAM1*	rs7105462	8.34 × 10^−15^	*NCAM1*	0.083	0.917	rs7105462*⁣*^*∗*^	0.084
IBS	Neuroticism	14q24.3	rs55707505	2.63 × 10^−11^	*DLST*	rs10144845	9.86 × 10^−12^	*YLPM1*	0.057	0.773	rs9671386	0.258
IBS	Neuroticism	22q13.2	rs5758268	1.05 × 10^−13^	*L3MBTL2*	rs3818003	1.19 × 10^−13^	*L3MBTL2*	0.088	0.908	rs2273085	0.123
UC	Neuroticism	3p21.1	rs1529544	1.14 × 10^−08^	*SFMBT1*	rs1529544	3.94 × 10^−08^	*SFMBT1*	0.067	0.896	rs1529544*⁣*^*∗*^	0.150
UC	Neuroticism	17q21.1	rs34003767	9.49 × 10^−10^	*MED24*	rs59716545	1.44 × 10^−10^	*ZPBP2*	0.067	0.907	rs35982947	0.124
IBS	Worry	1q21.3	rs11800001	9.54 × 10^−09^	*GATAD2B*	rs11800001	1.55 × 10^−08^	*GATAD2B*	0.087	0.751	rs11800001*⁣*^*∗*^	0.131
IBS	Worry	2q33.1	rs6738494	1.34 × 10^−08^	*FTCDNL1*	rs6738494	2.41 × 10^−08^	*FTCDNL1*	0.014	0.853	rs6738494*⁣*^*∗*^	0.205
IBS	Worry	3q22.3	rs1280624	1.62 × 10^−12^	*RP11-102M11.2*	rs1280624	9.71 × 10^−12^	*RP11-102M11.2*	0.166	0.784	rs1280624*⁣*^*∗*^	0.069
IBS	Worry	5q14.3	rs2195613	2.75 × 10^−10^	*TMEM161B-AS1*	rs7706932	3.57 × 10^−10^	*CTC-498M16.4*	0.067	0.778	rs7706932*⁣*^*∗*^	0.059
IBS	Worry	11q23.2	rs7947502	4.83 × 10^−14^	*NCAM1*	rs7947502	3.21 × 10^−13^	*NCAM1*	0.051	0.949	rs7947502*⁣*^*∗*^	0.225
IBS	Worry	22q13.2	rs136402	2.00 × 10^−11^	*L3MBTL2*	rs136402	2.93 × 10^−12^	*L3MBTL2*	0.141	0.851	rs136402*⁣*^*∗*^	0.055
UC	Worry	2q33.1	rs56322003	2.98 × 10^−11^	*AC018717.1*	rs62180151	3.24 × 10^−10^	*AC018717.1*	0.142	0.858	rs56322003*⁣*^*∗*^	0.0255
UC	Worry	17q21.1	rs34003767	4.10 × 10^−08^	*MED24*	rs59716545	1.39 × 10^−10^	*ZPBP2*	0.061	0.828	rs34003767*⁣*^*∗*^	0.125
PUD	Worry	2q24.3	rs79205304	2.06 × 10^−08^	*AC092684.1*	rs79205304	2.18 × 10^−08^	*AC092684.1*	0.072	0.802	rs75057174	0.091

IBD, inflammatory bowel disease; IBS, irritable bowel syndrome; PUD, peptic ulcer disease; GERD, gastroesophageal reflux disease; CD, Crohn's disease; UC, ulcerative colitis; PP.H3, posterior probability of H3; PP.H4, posterior probability of H4. *⁣*^*∗*^indicates that the causal SNV was also identified in FUMA, identified as a pleiotropic locus.

## Data Availability

All data, materials, and codes are available in the main text or the supplementary materials.

## Additional Points

*Limitations*. There were some limitations to our study. First, the data used included samples from the UK Biobank, with possible sample overlap. To account for this, we used methods robust for sample overlaps, such as the use of unrestricted intercept LDSC and the use of correlation matrices to decorrelate the data *z*-scores before using PLACO for the pleiotropic analysis. Second, the sample of GWAS data from the GIT diseases did not adequately exclude samples with mental disorders, which to some extent may explain some of the genetic overlaps. Our study has several advantages. We showed similarities in the shared genetic mechanisms of neurotic and psychotic disorders in the gut–brain axis, e.g., 11q23.2, and highlighted novel pleiotropic loci, e.g., 16q12.1 and 2q37.1. In addition, we showed genetic heterogeneity between neuroticism and different GIT diseases, e.g., 22q13.2 and 14q24.3, is pleiotropic only in IBS and neuroticism. *Transparency Declaration*. The manuscript is an honest, accurate, and transparent account of the study being reported. No important aspects of the study have been omitted. Any discrepancies from the study as planned (and, if relevant, registered) have been explained.

## Supplementary Materials

Supplementary 1 Figure 1: Manhattan plots of PLACO and MTAG results and shared loci by FUMA. Figure 2: locusCompare plots of 22 significantly colocalized loci. Figure 3: Expression of tissue-specific genes in 25 tissues. Figure 4: Forest plots of MR results.

Supplementary 2 Table 1: Details of GWAS Summary Data Sources. Table 2: TWAS analysis of the 25 specific tissues included and their code. Table 3: Genetic correlation between neuroticism and gastrointestinal tract diseases. Table 4: Summary of MTAG results. Table 5: Shared loci identified based on MTAG results using FUMA. Table 6: Shared loci identified based on PLACO results using FUMA. Table 7: Shared loci by comparing the FUMA results of MTAG and PLACO. Table 8: Lead SNPs by comparing the FUMA results of MTAG and PLACO. Table 9: Candidate pleiotropic genes identified by MAGMA. Table 10: Top 10 tissue-specific gene expression using JTI model. Table 11: instrumental variables for depression or dysthymia.
