# Supplementary material for: Shared and Unique Genetic Links between Neuroticism and Gastrointestinal Tract Diseases
Source: Depress Anxiety. 2024 Jun 21;2024:5515448. doi: 10.1155/2024/5515448 (PMC11919111; doi:10.1155/2024/5515448)
Supplement: Supplementary 2 — Table 1: Details of GWAS Summary Data Sources. Table 2: TWAS analysis of the 25 specific tissues included and their code. Table 3: Genetic correlation between neuroticism and gastrointestinal tract diseases. Table 4: Summary of MTAG results. Table 5: Shared loci identified based on MTAG results using FUMA. Table 6: Shared loci identified based on PLACO results using FUMA. Table 7: Shared loci by comparing the FUMA results of MTAG and PLACO. Table 8: Lead SNPs by comparing the FUMA results of MTAG and PLACO. Table 9: Candidate pleiotropic genes identified by MAGMA. Table 10: Top 10 tissue-specific gene expression using JTI model. Table 11: instrumental variables for depression or dysthymia. [file 5515448.f2.docx]

**Table S1**: Details of GWAS Summary Data Sources.

**Table S2**: TWAS analysis of the 25 specific tissues included and their code.

**Table S3**: Genetic correlation between neuroticism and gastrointestinal tract diseases

**Table S4**: Summary of MTAG results.

**Table S5**: Shared loci identified based on MTAG results using FUMA.

**Table S6**: Shared loci identified based on PLACO results using FUMA.

**Table S7**: The shared loci identified by comparing the FUMA results of MTAG and PLACO.

**Table S8**: The lead SNPs identified by comparing the FUMA results of MTAG and PLACO.

**Table S9**: Candidate Pleiotropic Genes Identified by MAGMA.

**Table S10**: Top 10 Tissue-specific gene expression using JTI model.

**Table S11**: Instrumental variables for depression or dysthymia.

**Table S1**: Details of GWAS Summary Data Sources

| **Neuroticism** | **PMID** | **GWAS data source** | **Adjustment** | **N** | **Ancestry** | **Genetic position** |
| --- | --- | --- | --- | --- | --- | --- |
| Depressed | 29942085 | UK Biobank | Ten genetic European-based principal components, age, sex, Townsend deprivation index, and,genotype array as covariates. | 357957 | European | GRCh37 |
| Neuroticism | 29942085 | UK Biobank and GPC1 (Exclude the 23andMe data) | Ten genetic European-based principal components, age, sex, Townsend deprivation index, and,genotype array as covariates. | 390278 | European | GRCh37 |
| Worry | 29942085 | UK Biobank | Ten genetic European-based principal components, age, sex, Townsend deprivation index, and,genotype array as covariates. | 348219 | European | GRCh37 |
| **Gastrointestinal Tract Diseases** | **PMID** | **GWAS data source** |  | **N** | **Ancestry** | **Genetic position** |
| Inflammatory bowel disease | 33608531 | UK Biobank | Sex, age and 20 ancestry principal components fitted as covariates. | 456327 | European | GRCh37 |
| Crohn's disease | 26192919 | non UK | The first 10 principal components as covariates. | 20883 | European | GRCh37 |
| Ulcerative colitis | 26192919 | non UK | The first 10 principal components as covariates. | 27432 | European | GRCh37 |
| Irritable bowel syndrome | 34741163 | UK Biobank | Sex, age and the first 20 principal components of the genetic data available through UK Biobank. | 486601 | European | GRCh37 |
| Peptic ulcer disease | 33608531 | UK Biobank | Sex, age and 20 ancestry principal components fitted as covariates. | 456327 | European | GRCh37 |
| Gastro-oesophageal reflux disease | 33608531 | UK Biobank | Sex, age and 20 ancestry principal components fitted as covariates. | 456327 | European | GRCh37 |

**Table S2**: TWAS analysis of the 25 specific tissues included and their code.

| **Tissue** | **Code** |
| --- | --- |
| Adrenal_Gland | T1 |
| Brain_Amygdala | T2 |
| Brain_Anterior_cingulate_cortex_BA24 | T3 |
| Brain_Caudate_basal_ganglia | T4 |
| Brain_Cerebellar_Hemisphere | T5 |
| Brain_Cerebellum | T6 |
| Brain_Cortex | T7 |
| Brain_Frontal_Cortex_BA9 | T8 |
| Brain_Hippocampus | T9 |
| Brain_Hypothalamus | T10 |
| Brain_Nucleus_accumbens_basal_ganglia | T11 |
| Brain_Putamen_basal_ganglia | T12 |
| Brain_Spinal_cord_cervical_c-1 | T13 |
| Brain_Substantia_nigra | T14 |
| Cells_EBV-transformed_lymphocytes | T15 |
| Colon_Sigmoid | T16 |
| Colon_Transverse | T17 |
| Esophagus_Gastroesophageal_Junction | T18 |
| Esophagus_Mucosa | T19 |
| Esophagus_Muscularis | T20 |
| Liver | T21 |
| Pituitary | T22 |
| Small_Intestine_Terminal_Ileum | T23 |
| Stomach | T24 |
| Whole_Blood | T25 |

**Table S3**: Genetic correlation between neuroticism and gastrointestinal tract diseases.

| **Trait1_category** | **Trait 1** | **Trait 2_category** | **Trait 2** | **LDSC** | | | **HDL** | | |
| --- | --- | --- | --- | --- | --- | --- | --- | --- | --- |
|  |  |  |  | **r_g_** | ***p*** | **se** | **r_g_** | ***p*** | **se** |
| Gastrointestinal disease | Inflammatory bowel disease | Negative emotion | Depressed | 0.092 | 8.74E-02 | 0.054 | 0.142 | 4.11E-08* | 0.026 |
| Gastrointestinal disease | Crohn's disease | Negative emotion | Depressed | 0.111 | 1.23E-02 | 0.044 | 0.087 | 3.75E-04* | 0.025 |
| Gastrointestinal disease | Ulcerative colitis | Negative emotion | Depressed | 0.067 | 1.79E-01 | 0.050 | 0.060 | 1.75E-02 | 0.025 |
| Gastrointestinal disease | Irritable bowel syndrome | Negative emotion | Depressed | 0.426 | 1.19E-10* | 0.066 | 0.492 | 3.71E-53* | 0.032 |
| Gastrointestinal disease | Peptic ulcer disease | Negative emotion | Depressed | 0.370 | 3.62E-18* | 0.043 | 0.402 | 7.19E-23* | 0.041 |
| Gastrointestinal disease | Gastroesophageal reflux disease | Negative emotion | Depressed | 0.480 | 1.03E-20* | 0.051 | 0.450 | 1.36E-65* | 0.026 |
| Gastrointestinal disease | Inflammatory bowel disease | Negative emotion | Neuroticism | 0.027 | 5.62E-01 | 0.047 | 0.088 | 3.58E-05* | 0.021 |
| Gastrointestinal disease | Crohn's disease | Negative emotion | Neuroticism | 0.083 | 3.21E-02 | 0.039 | 0.079 | 2.24E-04* | 0.021 |
| Gastrointestinal disease | Ulcerative colitis | Negative emotion | Neuroticism | 0.091 | 3.84E-02 | 0.044 | 0.101 | 2.65E-05* | 0.024 |
| Gastrointestinal disease | Irritable bowel syndrome | Negative emotion | Neuroticism | 0.415 | 3.79E-12* | 0.060 | 0.526 | 1.36E-67* | 0.030 |
| Gastrointestinal disease | Peptic ulcer disease | Negative emotion | Neuroticism | 0.270 | 7.93E-11* | 0.042 | 0.293 | 1.14E-14* | 0.038 |
| Gastrointestinal disease | Gastroesophageal reflux disease | Negative emotion | Neuroticism | 0.369 | 3.45E-16* | 0.045 | 0.363 | 1.36E-63* | 0.022 |
| Gastrointestinal disease | Inflammatory bowel disease | Negative emotion | Worry | 0.060 | 2.96E-01 | 0.057 | 0.032 | 1.97E-01 | 0.025 |
| Gastrointestinal disease | Crohn's disease | Negative emotion | Worry | 0.068 | 9.17E-02 | 0.040 | 0.070 | 2.30E-03 | 0.023 |
| Gastrointestinal disease | Ulcerative colitis | Negative emotion | Worry | 0.134 | 4.20E-03 | 0.047 | 0.158 | 1.10E-09* | 0.026 |
| Gastrointestinal disease | Irritable bowel syndrome | Negative emotion | Worry | 0.405 | 4.84E-10* | 0.065 | 0.506 | 1.20E-53* | 0.033 |
| Gastrointestinal disease | Peptic ulcer disease | Negative emotion | Worry | 0.160 | 1.00E-04* | 0.042 | 0.158 | 2.11E-06* | 0.033 |
| Gastrointestinal disease | Gastroesophageal reflux disease | Negative emotion | Worry | 0.255 | 1.49E-09* | 0.042 | 0.256 | 1.46E-33* | 0.021 |

Genetic correlation were estimated by LDSC and HDL methods, respectively. Bonferroni-corrected significance threshold was set at *p* < 2.78E-03 (0.05/18), producing a final union set of 16 pairwise traits with significant genetic correlation for subsequent analysis.

*indicates less than the Bonferroni-corrected significance threshold (2.78E-03).

**Table S4**: Summary of MTAG results.

| **Trait 1** | **Trait 2** | **MTAG result** | | |
| --- | --- | --- | --- | --- |
|  |  | **GWAS mean χ2** | **MTAG mean χ2** | **Max FDR** |
| Crohn's disease | Depressed | 1.151 | 1.153 | 0.031 |
| Gastrointestinal disease | Depressed | 1.234 | 1.28 | 0.006 |
| Irritable bowel syndrome | Depressed | 1.118 | 1.195 | 0.030 |
| Peptic ulcer disease | Depressed | 1.08 | 1.114 | 0.073* |
| Inflammatory bowel disease | Depressed | 1.084 | 1.094 | 0.100* |
| Crohn's disease | Neuroticism | 1.151 | 1.154 | 0.031 |
| Gastrointestinal disease | Neuroticism | 1.234 | 1.269 | 0.007 |
| Inflammatory bowel disease | Neuroticism | 1.084 | 1.091 | 0.110* |
| Irritable bowel syndrome | Neuroticism | 1.118 | 1.223 | 0.031 |
| Peptic ulcer disease | Neuroticism | 1.08 | 1.102 | 0.089* |
| Ulcerative colitis | Neuroticism | 1.09 | 1.095 | 0.097* |
| Gastrointestinal disease | Worry | 1.234 | 1.254 | 0.008 |
| Irritable bowel syndrome | Worry | 1.118 | 1.21 | 0.030 |
| Peptic ulcer disease | Worry | 1.08 | 1.088 | 0.109* |
| Ulcerative colitis | Worry | 1.09 | 1.104 | 0.080* |

*indicates maxFDR greater than 0.05,it may lead to inflation of the results, leading to false positive results.

**Table S5**: Shared loci identified based on MTAG results using FUMA

| **No.Genomic locus** | **Trait 1** | **Trait 2** | **Top SNP** | **CHR** | **POS** | **A1** | **A2** | ***p***MTAG | **Region** | **Mapped gene** |
| --- | --- | --- | --- | --- | --- | --- | --- | --- | --- | --- |
| 1 | CD | Depressed | rs697693 | 1 | 7886424 | A | G | 2.65E-09 | 1p36.23 | *VAMP3* |
| 2 | CD | Depressed | rs11209026 | 1 | 67705958 | A | G | 4.63E-52 | 1p31.3 | *IL23R* |
| 3 | CD | Depressed | rs1052177 | 1 | 155260350 | A | G | 2.26E-08 | 1q22 | *SCAMP3* |
| 4 | CD | Depressed | rs6704109 | 1 | 172857050 | C | T | 4.34E-15 | 1q24.3 | *AIMP1P2* |
| 5 | CD | Depressed | rs3024505 | 1 | 206939904 | A | G | 3.18E-09 | 1q32.1 | *Y_RNA/IL10* |
| 6 | CD | Depressed | rs7560838 | 2 | 43907630 | A | C | 1.31E-09 | 2p21 | *PLEKHH2* |
| 7 | CD | Depressed | rs112401990 | 2 | 61199327 | A | G | 1.09E-08 | 2p16.1 | *PUS10* |
| 8 | CD | Depressed | rs4851586 | 2 | 103064264 | C | T | 4.39E-10 | 2q12.1 | *IL18R1* |
| 9 | CD | Depressed | rs7423615 | 2 | 231116874 | C | T | 1.90E-08 | 2q37.1 | *SP140* |
| 10 | CD | Depressed | rs3816234 | 2 | 234185999 | A | G | 2.25E-37 | 2q37.1 | *ATG16L1/SCARNA5* |
| 11 | CD | Depressed | rs13096760 | 3 | 49476806 | C | T | 2.63E-12 | 3p21.31 | *NICN1/RNA5SP130* |
| 12 | CD | Depressed | rs13135092 | 4 | 103198082 | A | G | 2.49E-09 | 4q24 | *SLC39A8* |
| 13 | CD | Depressed | rs7713270 | 5 | 40440063 | C | T | 2.78E-34 | 5p13.1 | *TTC33* |
| 14 | CD | Depressed | rs2617439 | 5 | 96275154 | A | G | 9.58E-12 | 5q15 | *LNPEP* |
| 15 | CD | Depressed | rs2188962 | 5 | 131770805 | C | T | 4.27E-20 | 5q31.1 | *P4HA2/SLC22A4* |
| 16 | CD | Depressed | rs59624008 | 5 | 150235979 | C | T | 2.09E-17 | 5q33.1 | *IRGM* |
| 17 | CD | Depressed | rs10045431 | 5 | 158814533 | A | C | 5.79E-17 | 5q33.3 | *IL12B/LINC01845* |
| 18 | CD | Depressed | rs9350273 | 6 | 20721583 | A | C | 2.39E-08 | 6p22.3 | *CDKAL1* |
| 19 | CD | Depressed | rs28701841 | 6 | 106530330 | A | G | 4.00E-09 | 6q21 | *ATG5/PRDM1* |
| 20 | CD | Depressed | rs444210 | 6 | 167390242 | A | G | 1.75E-13 | 6q27 | *RNASET2/MIR3939* |
| 21 | CD | Depressed | rs1456896 | 7 | 50304461 | C | T | 1.98E-08 | 7p12.2 | *SPATA48/IKZF1* |
| 22 | CD | Depressed | rs921720 | 8 | 126534671 | A | G | 3.04E-12 | 8q24.13 | *TRIB1* |
| 23 | CD | Depressed | rs10758669 | 9 | 4981602 | A | C | 4.37E-10 | 9p24.1 | *HNRNPA1P41/JAK2* |
| 24 | CD | Depressed | rs3810936 | 9 | 117552885 | C | T | 5.17E-15 | 9q32 | *TNFSF15* |
| 25 | CD | Depressed | rs4077515 | 9 | 139266496 | C | T | 1.23E-19 | 9q34.3 | *CARD9* |
| 26 | CD | Depressed | rs2505640 | 10 | 35459497 | A | G | 4.89E-10 | 10p11.21 | *CREM* |
| 27 | CD | Depressed | rs10822050 | 10 | 64438771 | C | T | 1.54E-18 | 10q21.2 | *ALDH7A1P4* |
| 28 | CD | Depressed | rs1250573 | 10 | 81042475 | A | G | 1.57E-10 | 10q22.3 | *ZMIZ1* |
| 29 | CD | Depressed | rs1332099 | 10 | 101298451 | C | T | 1.68E-19 | 10q24.2 | *NKX2-3/SLC25A28* |
| 30 | CD | Depressed | rs11236791 | 11 | 76295598 | A | G | 2.77E-15 | 11q13.5 | *EMSY/LINC02757* |
| 31 | CD | Depressed | rs140892874 | 12 | 40824798 | C | T | 2.69E-17 | 12q12 | *MUC19* |
| 32 | CD | Depressed | rs1932990 | 13 | 44460242 | C | T | 6.02E-09 | 13q14.11 | *LACC1* |
| 33 | CD | Depressed | rs56062135 | 15 | 67455630 | C | T | 3.12E-12 | 15q22.33 | *SMAD3* |
| 34 | CD | Depressed | rs4788084 | 16 | 28539848 | C | T | 3.97E-10 | 16p11.2 | *IL27/NUPR1* |
| 35 | CD | Depressed | rs75294107 | 16 | 49671101 | A | G | 1.03E-08 | 16q12.1 | *ZNF423* |
| 36 | CD | Depressed | rs2076756 | 16 | 50756881 | A | G | 2.28E-59 | 16q12.1 | *NOD2* |
| 37 | CD | Depressed | rs3091315 | 17 | 32593665 | A | G | 1.30E-11 | 17q12 | *CCL2/CCL7* |
| 38 | CD | Depressed | rs12936409 | 17 | 38043649 | C | T | 4.82E-09 | 17q21.1 | *ZPBP2/GSDMB* |
| 39 | CD | Depressed | rs80262450 | 18 | 12818922 | A | G | 3.17E-15 | 18p11.21 | *PTPN2* |
| 40 | CD | Depressed | rs11669443 | 19 | 1109215 | A | G | 6.86E-12 | 19p13.3 | *SBNO2* |
| 41 | CD | Depressed | rs142770866 | 19 | 10525372 | A | G | 1.12E-08 | 19p13.2 | *CDC37* |
| 42 | CD | Depressed | rs2638281 | 19 | 49210869 | A | G | 6.26E-09 | 19q13.33 | *FUT2* |
| 43 | CD | Depressed | rs1056441 | 20 | 62370349 | C | T | 6.77E-11 | 20q13.33 | *LIME1* |
| 44 | CD | Depressed | rs1297271 | 21 | 16823163 | C | T | 1.79E-10 | 21q21.1 | *CYCSP42* |
| 45 | CD | Depressed | rs7276302 | 21 | 45614159 | A | G | 1.82E-13 | 21q22.3 | *GATD3A* |
| 46 | CD | Depressed | rs4820091 | 22 | 21940189 | G | T | 5.73E-09 | 22q11.21 | *UBE2L3* |
| 47 | CD | Depressed | rs151314883 | 22 | 39735087 | A | G | 5.12E-11 | 22q13.1 | *RPL3/SYNGR1* |
| 48 | GERD | Depressed | rs66595136 | 2 | 64147917 | A | G | 7.34E-09 | 2p15 | *WDPCP* |
| 49 | GERD | Depressed | rs1430788 | 2 | 67868412 | C | T | 1.23E-09 | 2p14 | *LINC02831* |
| 50 | GERD | Depressed | rs73079003 | 3 | 49832788 | A | G | 5.57E-10 | 3p21.31 | *CDHR4* |
| 51 | GERD | Depressed | rs13135092 | 4 | 103198082 | A | G | 2.25E-08 | 4q24 | *SLC39A8* |
| 52 | GERD | Depressed | rs32546 | 5 | 13205087 | C | T | 3.69E-08 | 5p15.2 | *RPS23P5* |
| 53 | GERD | Depressed | rs7752448 | 6 | 28301099 | A | G | 3.39E-13 | 6p22.1 | *ZSCAN31* |
| 54 | GERD | Depressed | rs1940722 | 11 | 112856359 | C | T | 1.65E-11 | 11q23.2 | *NCAM1* |
| 55 | GERD | Depressed | rs11652896 | 17 | 11477247 | C | T | 2.09E-08 | 17p12 | *SHISA6* |
| 56 | GERD | Depressed | rs967823 | 17 | 50317276 | A | G | 1.43E-08 | 17q22 | *CA10* |
| 57 | IBD | Depressed | rs10737482 | 1 | 20173858 | C | T | 6.51E-15 | 1p36.13 | *RNF186/OTUD3* |
| 58 | IBD | Depressed | rs11581607 | 1 | 67707690 | A | G | 1.41E-19 | 1p31.3 | *IL23R* |
| 59 | IBD | Depressed | rs6671847 | 1 | 161478810 | A | G | 9.31E-11 | 1q23.3 | *FCGR2A* |
| 60 | IBD | Depressed | rs905634 | 1 | 200884985 | C | T | 2.62E-11 | 1q32.1 | *INAVA/MROH3P* |
| 61 | IBD | Depressed | rs3024493 | 1 | 206943968 | A | C | 4.91E-14 | 1q32.1 | *IL10* |
| 62 | IBD | Depressed | rs7562347 | 2 | 61209875 | G | T | 2.79E-08 | 2p16.1 | *PUS10* |
| 63 | IBD | Depressed | rs13384671 | 2 | 182311594 | A | G | 4.23E-08 | 2q31.3 | *LINC01934/ITGA4* |
| 64 | IBD | Depressed | rs348594 | 5 | 40323938 | A | G | 2.01E-09 | 5p13.1 | *LOC105374736* |
| 65 | IBD | Depressed | rs17264332 | 6 | 138005515 | A | G | 1.27E-09 | 6q23.3 | *SLC17A1/LINC02539* |
| 66 | IBD | Depressed | rs6964893 | 7 | 107499725 | G | T | 2.18E-08 | 7q31.1 | *PIGCP2/DLD* |
| 67 | IBD | Depressed | rs3757387 | 7 | 128576086 | C | T | 2.17E-08 | 7q32.1 | *KCP/IRF5* |
| 68 | IBD | Depressed | rs10761659 | 10 | 64445564 | A | G | 4.90E-10 | 10q21.2 | *ALDH7A1P4* |
| 69 | IBD | Depressed | rs7095491 | 10 | 101274058 | C | T | 8.26E-10 | 10q24.2 | *GOT1/LINC01475* |
| 70 | IBD | Depressed | rs2212434 | 11 | 76281593 | C | T | 5.10E-11 | 11q13.5 | *EMSY/LINC02757* |
| 71 | IBD | Depressed | rs11614178 | 12 | 68508122 | A | G | 1.29E-08 | 12q15 | *IFNG-AS1* |
| 72 | IBD | Depressed | rs12720356 | 19 | 10469975 | A | C | 4.24E-08 | 19p13.2 | *TYK2* |
| 73 | IBD | Depressed | rs6017342 | 20 | 43065028 | A | C | 7.74E-12 | 20q13.12 | *LINC01620* |
| 74 | IBD | Depressed | rs1297261 | 21 | 16812623 | C | T | 1.46E-10 | 21q21.1 | *CYCSP42* |
| 75 | IBD | Depressed | rs2836878 | 21 | 40465534 | A | G | 9.61E-19 | 21q22.2 | *RPL23AP12* |
| 76 | IBD | Depressed | rs2838517 | 21 | 45613825 | C | T | 1.02E-08 | 21q22.3 | *GATD3A* |
| 77 | IBS | Depressed | rs301806 | 1 | 8482078 | C | T | 8.94E-09 | 1p36.23 | *RERE* |
| 78 | IBS | Depressed | rs1609391 | 3 | 136615268 | A | G | 2.31E-08 | 3q22.3 | *NCK1* |
| 79 | IBS | Depressed | rs3807866 | 7 | 12250378 | A | G | 1.78E-09 | 7p21.3 | *TMEM106B* |
| 80 | IBS | Depressed | rs10821129 | 9 | 96222617 | A | G | 5.23E-10 | 9q22.31 | *FAM120A* |
| 81 | IBS | Depressed | rs10891490 | 11 | 112885527 | C | T | 3.47E-12 | 11q23.2 | *NCAM1* |
| 82 | IBS | Depressed | rs4245154 | 11 | 113388674 | A | G | 7.48E-09 | 11q23.2 | *DRD2* |
| 83 | IBS | Depressed | rs10144845 | 14 | 75237770 | C | T | 2.03E-10 | 14q24.3 | *YLPM1* |
| 84 | IBS | Depressed | rs11665070 | 18 | 35152563 | A | G | 1.52E-10 | 18q12.2 | *CELF4/MIR4318* |
| 85 | IBS | Depressed | rs136402 | 22 | 41598933 | A | G | 1.45E-09 | 22q13.2 | *EP300-AS1/L3MBTL2* |
| 86 | PUD | Depressed | rs2976387 | 8 | 143759364 | A | G | 8.21E-12 | 8q24.3 | *PSCA/JRK* |
| 87 | PUD | Depressed | rs10500661 | 11 | 6273744 | C | T | 4.14E-10 | 11p15.4 | *CNGA4/CCKBR* |
| 88 | PUD | Depressed | rs613872 | 18 | 53210302 | G | T | 4.27E-09 | 18q21.2 | *TCF4* |
| 89 | PUD | Depressed | rs681343 | 19 | 49206462 | C | T | 5.50E-11 | 19q13.33 | *FUT2* |
| 90 | CD | Neuroticism | rs697693 | 1 | 7886424 | A | G | 2.95E-09 | 1p36.23 | *PER3* |
| 91 | CD | Neuroticism | rs11209026 | 1 | 67705958 | A | G | 3.35E-52 | 1p31.3 | *IL23R* |
| 92 | CD | Neuroticism | rs6704109 | 1 | 172857050 | C | T | 9.44E-15 | 1q24.3 | *AIMP1P2* |
| 93 | CD | Neuroticism | rs3024505 | 1 | 206939904 | A | G | 2.85E-09 | 1q32.1 | *Y_RNA/IL10* |
| 94 | CD | Neuroticism | rs10186441 | 2 | 43840048 | A | G | 9.05E-10 | 2p21 | *Y_RNA/PLEKHH2* |
| 95 | CD | Neuroticism | rs112401990 | 2 | 61199327 | A | G | 6.61E-09 | 2p16.1 | *PUS10* |
| 96 | CD | Neuroticism | rs4851586 | 2 | 103064264 | C | T | 8.67E-10 | 2q12.1 | *IL18RAP* |
| 97 | CD | Neuroticism | rs7423615 | 2 | 231116874 | C | T | 1.67E-08 | 2q37.1 | *SP140* |
| 98 | CD | Neuroticism | rs3816234 | 2 | 234185999 | A | G | 9.40E-38 | 2q37.1 | *ATG16L1* |
| 99 | CD | Neuroticism | rs13096760 | 3 | 49476806 | C | T | 1.34E-12 | 3p21.31 | *USP4* |
| 100 | CD | Neuroticism | rs13135092 | 4 | 103198082 | A | G | 2.60E-09 | 4q24 | *SLC39A8* |
| 101 | CD | Neuroticism | rs7713270 | 5 | 40440063 | C | T | 4.20E-34 | 5p13.1 | *TTC33* |
| 102 | CD | Neuroticism | rs2617439 | 5 | 96275154 | A | G | 1.95E-11 | 5q15 | *LNPEP* |
| 103 | CD | Neuroticism | rs2188962 | 5 | 131770805 | C | T | 4.52E-20 | 5q31.1 | *IRF1-AS1* |
| 104 | CD | Neuroticism | rs59624008 | 5 | 150235979 | C | T | 2.25E-17 | 5q33.1 | *ZNF300/IRGM* |
| 105 | CD | Neuroticism | rs10045431 | 5 | 158814533 | A | C | 2.44E-16 | 5q33.3 | *IL12B/LINC01845* |
| 106 | CD | Neuroticism | rs9350273 | 6 | 20721583 | A | C | 1.23E-08 | 6p22.3 | *CDKAL1* |
| 107 | CD | Neuroticism | rs28701841 | 6 | 106530330 | A | G | 3.27E-09 | 6q21 | *ATG5/PRDM1* |
| 108 | CD | Neuroticism | rs444210 | 6 | 167390242 | A | G | 7.21E-14 | 6q27 | *RNASET2/MIR3939* |
| 109 | CD | Neuroticism | rs921720 | 8 | 126534671 | A | G | 3.80E-12 | 8q24.13 | *TRIB1* |
| 110 | CD | Neuroticism | rs10758669 | 9 | 4981602 | A | C | 4.42E-10 | 9p24.1 | *HNRNPA1P41/JAK2* |
| 111 | CD | Neuroticism | rs3810936 | 9 | 117552885 | C | T | 5.47E-15 | 9q32 | *TNFSF15* |
| 112 | CD | Neuroticism | rs4077515 | 9 | 139266496 | C | T | 1.06E-19 | 9q34.3 | *CARD9* |
| 113 | CD | Neuroticism | rs2505640 | 10 | 35459497 | A | G | 2.52E-10 | 10p11.21 | *CREM* |
| 114 | CD | Neuroticism | rs12766391 | 10 | 64441204 | A | G | 1.87E-18 | 10q21.2 | *ZNF365/ALDH7A1P4* |
| 115 | CD | Neuroticism | rs1250573 | 10 | 81042475 | A | G | 2.17E-10 | 10q22.3 | *ZMIZ1* |
| 116 | CD | Neuroticism | rs1332099 | 10 | 101298451 | C | T | 4.17E-19 | 10q24.2 | *NKX2-3/SLC25A28* |
| 117 | CD | Neuroticism | rs11236791 | 11 | 76295598 | A | G | 3.51E-15 | 11q13.5 | *EMSY/WLINC02757* |
| 118 | CD | Neuroticism | rs140892874 | 12 | 40824798 | C | T | 2.69E-17 | 12q12 | *MUC19* |
| 119 | CD | Neuroticism | rs1932990 | 13 | 44460242 | C | T | 2.86E-09 | 13q14.11 | *LACC1/NRAD1* |
| 120 | CD | Neuroticism | rs17228058 | 15 | 67450305 | A | G | 3.43E-12 | 15q22.33 | *SMAD3* |
| 121 | CD | Neuroticism | rs4788084 | 16 | 28539848 | C | T | 5.06E-10 | 16p11.2 | *IL27 - NUPR1* |
| 122 | CD | Neuroticism | rs75294107 | 16 | 49671101 | A | G | 1.26E-08 | 16q12.1 | *ZNF423* |
| 123 | CD | Neuroticism | rs2076756 | 16 | 50756881 | A | G | 1.37E-59 | 16q12.1 | *NOD2* |
| 124 | CD | Neuroticism | rs3091315 | 17 | 32593665 | A | G | 1.45E-11 | 17q12 | *CCL2/CCL7* |
| 125 | CD | Neuroticism | rs12936409 | 17 | 38043649 | C | T | 4.22E-09 | 17q21.1 | *ZPBP2/GSDMB* |
| 126 | CD | Neuroticism | rs80262450 | 18 | 12818922 | A | G | 1.45E-15 | 18p11.21 | *PTPN2* |
| 127 | CD | Neuroticism | rs11669443 | 19 | 1109215 | A | G | 5.89E-12 | 19p13.3 | *SBNO2* |
| 128 | CD | Neuroticism | rs11669299 | 19 | 10496621 | C | T | 1.72E-08 | 19p13.2 | *CDC37* |
| 129 | CD | Neuroticism | rs281379 | 19 | 49214274 | A | G | 4.46E-09 | 19q13.33 | *FUT2/MAMSTR* |
| 130 | CD | Neuroticism | rs1056441 | 20 | 62370349 | C | T | 2.05E-10 | 20q13.33 | *LIME1* |
| 131 | CD | Neuroticism | rs1297271 | 21 | 16823163 | C | T | 1.25E-10 | 21q21.1 | *CYCSP42* |
| 132 | CD | Neuroticism | rs7276302 | 21 | 45614159 | A | G | 2.53E-13 | 21q22.3 | *GATD3A* |
| 133 | CD | Neuroticism | rs4820091 | 22 | 21940189 | G | T | 5.31E-09 | 22q11.21 | *UBE2L3* |
| 134 | CD | Neuroticism | rs151314883 | 22 | 39735087 | A | G | 3.00E-11 | 22q13.1 | *RPL3/SYNGR1* |
| 135 | GERD | Neuroticism | rs66595136 | 2 | 64147917 | A | G | 1.80E-09 | 2p15 | *WDPCP* |
| 136 | GERD | Neuroticism | rs1430788 | 2 | 67868412 | C | T | 8.23E-09 | 2p14 | *LINC02831* |
| 137 | GERD | Neuroticism | rs1858828 | 3 | 50242387 | G | T | 3.31E-08 | 3p21.31 | *SEMA3F* |
| 138 | GERD | Neuroticism | rs7752448 | 6 | 28301099 | A | G | 2.80E-14 | 6p22.1 | *ZSCAN31* |
| 139 | GERD | Neuroticism | rs4236599 | 7 | 114318097 | C | T | 2.34E-08 | 7q31.1 | *FOXP2* |
| 140 | GERD | Neuroticism | rs1940722 | 11 | 112856359 | C | T | 1.53E-11 | 11q23.2 | *NCAM1* |
| 141 | GERD | Neuroticism | rs967823 | 17 | 50317276 | A | G | 1.92E-09 | 17q22 | *CA10* |
| 142 | GERD | Neuroticism | rs11665070 | 18 | 35152563 | A | G | 4.94E-08 | 18q12.2 | *CELF4/MIR4318* |
| 143 | IBD | Neuroticism | rs6426832 | 1 | 20166458 | A | G | 7.97E-15 | 1p36.13 | *RNF186/OTUD3* |
| 144 | IBD | Neuroticism | rs11799474 | 1 | 22704191 | G | T | 3.97E-08 | 1p36.12 | *PPIAP34/ZBTB40* |
| 145 | IBD | Neuroticism | rs11581607 | 1 | 67707690 | A | G | 4.86E-21 | 1p31.3 | *IL23R* |
| 146 | IBD | Neuroticism | rs11204894 | 1 | 151793174 | G | T | 4.86E-08 | 1q21.3 | *RORC* |
| 147 | IBD | Neuroticism | rs6671847 | 1 | 161478810 | A | G | 1.38E-10 | 1q23.3 | *FCGR2A* |
| 148 | IBD | Neuroticism | rs905634 | 1 | 200884985 | C | T | 2.79E-12 | 1q32.1 | *INAVA/MROH3P* |
| 149 | IBD | Neuroticism | rs3024505 | 1 | 206939904 | A | G | 2.97E-14 | 1q32.1 | *Y_RNA/IL10* |
| 150 | IBD | Neuroticism | rs10188217 | 2 | 61217542 | C | T | 1.11E-08 | 2p16.1 | *PUS10* |
| 151 | IBD | Neuroticism | rs13384671 | 2 | 182311594 | A | G | 2.21E-08 | 2q31.3 | *LINC01934/ITGA4* |
| 152 | IBD | Neuroticism | rs7713270 | 5 | 40440063 | C | T | 2.60E-09 | 5p13.1 | *TTC33* |
| 153 | IBD | Neuroticism | rs55722650 | 5 | 131607300 | C | T | 3.81E-08 | 5q31.1 | *P4HA2/PDLIM4* |
| 154 | IBD | Neuroticism | rs17264332 | 6 | 138005515 | A | G | 1.26E-08 | 6q23.3 | *SLC17A1/LINC02539* |
| 155 | IBD | Neuroticism | rs6961243 | 7 | 107521404 | A | G | 2.11E-08 | 7q31.1 | *PIGCP2/DLD* |
| 156 | IBD | Neuroticism | rs3757387 | 7 | 128576086 | C | T | 4.29E-08 | 7q32.1 | *KCP/IRF5* |
| 157 | IBD | Neuroticism | rs10761659 | 10 | 64445564 | A | G | 6.20E-10 | 10q21.2 | *ALDH7A1P4* |
| 158 | IBD | Neuroticism | rs7095491 | 10 | 101274058 | C | T | 1.49E-09 | 10q24.2 | *NKX2-3/SLC25A28* |
| 159 | IBD | Neuroticism | rs2212434 | 11 | 76281593 | C | T | 7.28E-11 | 11q13.5 | *EMSY/LINC02757* |
| 160 | IBD | Neuroticism | rs12810476 | 12 | 68481756 | C | T | 1.82E-08 | 12q15 | *IFNG-AS1* |
| 161 | IBD | Neuroticism | rs6017342 | 20 | 43065028 | A | C | 2.30E-12 | 20q13.12 | *LINC01620* |
| 162 | IBD | Neuroticism | rs1297261 | 21 | 16812623 | C | T | 2.38E-11 | 21q21.1 | *CYCSP42* |
| 163 | IBD | Neuroticism | rs4817987 | 21 | 40465901 | C | T | 1.61E-18 | 21q22.2 | *RPL23AP12* |
| 164 | IBD | Neuroticism | rs2838516 | 21 | 45612475 | A | C | 1.05E-08 | 21q22.3 | *GATD3A* |
| 165 | IBS | Neuroticism | rs301806 | 1 | 8482078 | C | T | 4.23E-08 | 1p36.23 | *RERE* |
| 166 | IBS | Neuroticism | rs75701938 | 3 | 107172033 | A | C | 4.21E-08 | 3q13.12 | *LINC01990* |
| 167 | IBS | Neuroticism | rs1729951 | 3 | 136500733 | G | T | 1.73E-11 | 3q22.3 | *PPP2R3A* |
| 168 | IBS | Neuroticism | rs7706932 | 5 | 87775691 | C | T | 2.97E-09 | 5q14.3 | *TMEM161B-DT/LINC00461* |
| 169 | IBS | Neuroticism | rs3807866 | 7 | 12250378 | A | G | 8.19E-09 | 7p21.3 | *TMEM106B* |
| 170 | IBS | Neuroticism | rs2721940 | 8 | 116636281 | A | C | 4.61E-08 | 8q23.3 | *TRPS1* |
| 171 | IBS | Neuroticism | rs10821129 | 9 | 96222617 | A | G | 6.33E-11 | 9q22.31 | *FAM120A* |
| 172 | IBS | Neuroticism | rs7034482 | 9 | 120500533 | A | G | 8.25E-09 | 9q33.1 | *TLR4/TPT1P9* |
| 173 | IBS | Neuroticism | rs7105462 | 11 | 112912048 | A | G | 8.34E-15 | 11q23.2 | *NCAM1* |
| 174 | IBS | Neuroticism | rs7111031 | 11 | 113377488 | A | C | 9.00E-11 | 11q23.2 | *DRD2/TMPRSS5* |
| 175 | IBS | Neuroticism | rs10144845 | 14 | 75237770 | C | T | 9.86E-12 | 14q24.3 | *YLPM1* |
| 176 | IBS | Neuroticism | rs77804065 | 17 | 43810896 | C | T | 1.09E-10 | 17q21.31 | *LINC02210-CRHR1* |
| 177 | IBS | Neuroticism | rs11665070 | 18 | 35152563 | A | G | 2.17E-14 | 18q12.2 | *CELF4* |
| 178 | IBS | Neuroticism | rs3818003 | 22 | 41609690 | C | T | 1.19E-13 | 22q13.2 | *L3MBTL2-AS1/L3MBTL2* |
| 179 | PUD | Neuroticism | rs2976387 | 8 | 143759364 | A | G | 3.84E-13 | 8q24.3 | *PSCA* |
| 180 | PUD | Neuroticism | rs687621 | 9 | 136137065 | A | G | 6.16E-09 | 9q34.2 | *ABO* |
| 181 | PUD | Neuroticism | rs78459074 | 11 | 1029905 | A | G | 3.77E-08 | 11p15.5 | *MUC6* |
| 182 | PUD | Neuroticism | rs10500661 | 11 | 6273744 | C | T | 2.61E-10 | 11p15.4 | *CNGA4/CCKBR* |
| 183 | PUD | Neuroticism | rs34074411 | 17 | 39867248 | C | T | 4.28E-08 | 17q21.2 | *EIF1/GAST* |
| 184 | PUD | Neuroticism | rs681343 | 19 | 49206462 | C | T | 3.75E-13 | 19q13.33 | *FUT2* |
| 185 | UC | Neuroticism | rs1886731 | 1 | 2472081 | C | T | 3.66E-10 | 1p36.32 | *TNFRSF14* |
| 186 | UC | Neuroticism | rs7523335 | 1 | 8180210 | A | G | 1.26E-09 | 1p36.23 | *PARK7* |
| 187 | UC | Neuroticism | rs10737481 | 1 | 20171514 | G | T | 2.74E-29 | 1p36.13 | *RNF186/OTUD3* |
| 188 | UC | Neuroticism | rs34920465 | 1 | 22700351 | A | G | 5.67E-11 | 1p36.12 | *PPIAP34/ZBTB40* |
| 189 | UC | Neuroticism | rs11581607 | 1 | 67707690 | A | G | 2.43E-24 | 1p31.3 | *IL23R* |
| 190 | UC | Neuroticism | rs1801274 | 1 | 161479745 | A | G | 8.10E-15 | 1q23.3 | *FCGR2A* |
| 191 | UC | Neuroticism | rs41299637 | 1 | 200877850 | G | T | 3.59E-11 | 1q32.1 | *INAVA* |
| 192 | UC | Neuroticism | rs3024493 | 1 | 206943968 | A | C | 4.51E-17 | 1q32.1 | *IL10* |
| 193 | UC | Neuroticism | rs10182512 | 2 | 61189469 | A | G | 8.80E-14 | 2p16.1 | *PUS10* |
| 194 | UC | Neuroticism | rs62180151 | 2 | 199699020 | G | T | 2.62E-08 | 2q33.1 | *SATB2* |
| 195 | UC | Neuroticism | rs4676410 | 2 | 241563739 | A | G | 1.32E-11 | 2q37.3 | *GPR35* |
| 196 | UC | Neuroticism | rs1131095 | 3 | 49714225 | C | T | 1.50E-12 | 3p21.31 | *APEH* |
| 197 | UC | Neuroticism | rs1529544 | 3 | 53039455 | A | G | 3.94E-08 | 3p21.1 | *SFMBT1* |
| 198 | UC | Neuroticism | rs34112392 | 5 | 149616316 | C | T | 1.82E-08 | 5q32 | *CAMK2A* |
| 199 | UC | Neuroticism | rs983825 | 5 | 158774965 | A | C | 1.04E-09 | 5q33.3 | *IL12B* |
| 200 | UC | Neuroticism | rs7752873 | 6 | 106579332 | C | T | 1.84E-08 | 6q21 | *ATG5* |
| 201 | UC | Neuroticism | rs62432712 | 6 | 137964697 | A | G | 1.37E-10 | 6q23.3 | *BTF3L4P3/LINC02539* |
| 202 | UC | Neuroticism | rs798502 | 7 | 2789880 | A | C | 8.40E-09 | 7p22.3 | *GNA12/AMZ1* |
| 203 | UC | Neuroticism | rs4380874 | 7 | 107480315 | C | T | 7.31E-15 | 7q31.1 | *PIGCP2/DLD* |
| 204 | UC | Neuroticism | rs10758669 | 9 | 4981602 | A | C | 9.82E-12 | 9p24.1 | *HNRNPA1P41/JAK2* |
| 205 | UC | Neuroticism | rs3812565 | 9 | 139272502 | C | T | 1.97E-12 | 9q34.3 | *SNAPC4* |
| 206 | UC | Neuroticism | rs6584283 | 10 | 101290301 | C | T | 3.00E-14 | 10q24.2 | *LINC01475* |
| 207 | UC | Neuroticism | rs2212434 | 11 | 76281593 | C | T | 2.22E-11 | 11q13.5 | *EMSY/LINC02757* |
| 208 | UC | Neuroticism | rs477238 | 11 | 114324026 | A | G | 6.90E-09 | 11q23.2 | *REXO2/NXPE2P1* |
| 209 | UC | Neuroticism | rs12817473 | 12 | 68497408 | A | G | 4.03E-15 | 12q15 | *IFNG-AS1* |
| 210 | UC | Neuroticism | rs1359946 | 13 | 27536972 | A | G | 1.63E-09 | 13q12.13 | *FGFR1OP2P1/RPS21P8* |
| 211 | UC | Neuroticism | rs59716545 | 17 | 38031857 | G | T | 1.44E-10 | 17q21.1 | *ZPBP2* |
| 212 | UC | Neuroticism | rs6017342 | 20 | 43065028 | A | C | 1.25E-13 | 20q13.12 | *LINC01620* |
| 213 | UC | Neuroticism | rs6062496 | 20 | 62329099 | A | G | 2.59E-10 | 20q13.33 | *RTEL1-TNFRSF6B/TNFRSF6B* |
| 214 | UC | Neuroticism | rs2836882 | 21 | 40466570 | A | G | 1.48E-18 | 21q22.2 | *RPL23AP12* |
| 215 | UC | Neuroticism | rs7282490 | 21 | 45615741 | A | G | 6.29E-10 | 21q22.3 | *GATD3A* |
| 216 | GERD | Worry | rs66595136 | 2 | 64147917 | A | G | 9.88E-09 | 2p15 | *WDPCP* |
| 217 | GERD | Worry | rs7752448 | 6 | 28301099 | A | G | 6.40E-14 | 6p22.1 | *ZSCAN31* |
| 218 | GERD | Worry | rs2186874 | 11 | 112912724 | C | T | 1.05E-09 | 11q23.2 | *NCAM1* |
| 219 | GERD | Worry | rs967823 | 17 | 50317276 | A | G | 3.10E-09 | 17q22 | *CA10* |
| 220 | IBS | Worry | rs11800001 | 1 | 153870850 | C | T | 1.55E-08 | 1q21.3 | *SLC39A1* |
| 221 | IBS | Worry | rs6738494 | 2 | 200697058 | A | C | 2.41E-08 | 2q33.1 | *FTCDNL1* |
| 222 | IBS | Worry | rs352140 | 3 | 52256697 | C | T | 4.71E-09 | 3p21.2 | *TLR9* |
| 223 | IBS | Worry | rs62250713 | 3 | 85513793 | A | G | 3.92E-13 | 3p12.1 | *CADM2* |
| 224 | IBS | Worry | rs1280624 | 3 | 136496690 | C | T | 9.71E-12 | 3q22.3 | *STAG1* |
| 225 | IBS | Worry | rs7706932 | 5 | 87775691 | C | T | 3.57E-10 | 5q14.3 | *TMEM161B-DT/LINC00461* |
| 226 | IBS | Worry | rs10992744 | 9 | 96230194 | A | G | 4.09E-10 | 9q22.31 | *FAM120A* |
| 227 | IBS | Worry | rs11191424 | 10 | 104625886 | A | G | 6.21E-09 | 10q24.32 | *BORCS7-ASMT* |
| 228 | IBS | Worry | rs7947502 | 11 | 112909396 | C | T | 3.21E-13 | 11q23.2 | *NCAM1* |
| 229 | IBS | Worry | rs9527336 | 13 | 55783042 | A | G | 3.59E-08 | 13q21.1 | *MIR5007* |
| 230 | IBS | Worry | rs55707505 | 14 | 75362552 | C | T | 2.07E-11 | 14q24.3 | *DLST* |
| 231 | IBS | Worry | rs11665070 | 18 | 35152563 | A | G | 4.83E-10 | 18q12.2 | *CELF4 - MIR4318* |
| 232 | IBS | Worry | rs2168711 | 18 | 57848531 | C | T | 1.59E-09 | 18q21.32 | *RNU4-17P/MC4R* |
| 233 | IBS | Worry | rs136402 | 22 | 41598933 | A | G | 2.93E-12 | 22q13.2 | *EP300-AS1/L3MBTL2* |
| 234 | PUD | Worry | rs6694256 | 1 | 155106768 | C | T | 1.71E-08 | 1q22 | *EFN1/SLC50A1* |
| 235 | PUD | Worry | rs79205304 | 2 | 164600989 | A | C | 2.18E-08 | 2q24.3 | *FIGN* |
| 236 | PUD | Worry | rs2976387 | 8 | 143759364 | A | G | 6.31E-14 | 8q24.3 | *PSCA* |
| 237 | PUD | Worry | rs687621 | 9 | 136137065 | A | G | 1.87E-09 | 9q34.2 | *ABO* |
| 238 | PUD | Worry | rs78459074 | 11 | 1029905 | A | G | 2.40E-10 | 11p15.5 | *MUC6* |
| 239 | PUD | Worry | rs10500661 | 11 | 6273744 | C | T | 5.44E-12 | 11p15.4 | *CNGA4/CCKBR* |
| 240 | PUD | Worry | rs9581957 | 13 | 28557889 | C | T | 1.27E-08 | 13q12.2 | *URAD* |
| 241 | PUD | Worry | rs34074411 | 17 | 39867248 | C | T | 4.55E-09 | 17q21.2 | *EIF1/GAST* |
| 242 | PUD | Worry | rs681343 | 19 | 49206462 | C | T | 5.99E-14 | 19q13.33 | *FUT2* |
| 243 | UC | Worry | rs2495366 | 1 | 2488930 | A | G | 8.23E-10 | 1p36.32 | *TNFRSF14* |
| 244 | UC | Worry | rs7523335 | 1 | 8180210 | A | G | 2.71E-09 | 1p36.23 | *PARK7* |
| 245 | UC | Worry | rs10737481 | 1 | 20171514 | G | T | 1.70E-24 | 1p36.13 | *RNF186/OTUD3* |
| 246 | UC | Worry | rs34920465 | 1 | 22700351 | A | G | 3.94E-10 | 1p36.12 | *PPIAP34/ZBTB40* |
| 247 | UC | Worry | rs11209026 | 1 | 67705958 | A | G | 1.92E-22 | 1p31.3 | *IL23R* |
| 248 | UC | Worry | rs1801274 | 1 | 161479745 | A | G | 1.28E-13 | 1q23.3 | *FCGR2A* |
| 249 | UC | Worry | rs41299637 | 1 | 200877850 | G | T | 1.61E-10 | 1q32.1 | *INAVA* |
| 250 | UC | Worry | rs3024493 | 1 | 206943968 | A | C | 1.84E-14 | 1q32.1 | *IL10* |
| 251 | UC | Worry | rs10182512 | 2 | 61189469 | A | G | 3.60E-14 | 2p16.1 | *PUS10* |
| 252 | UC | Worry | rs62180151 | 2 | 199699020 | G | T | 3.24E-10 | 2q33.1 | *PKP4* |
| 253 | UC | Worry | rs4676410 | 2 | 241563739 | A | G | 8.23E-11 | 2q37.3 | *GPR35* |
| 254 | UC | Worry | rs11130213 | 3 | 49712297 | C | T | 1.34E-12 | 3p21.31 | *APEH* |
| 255 | UC | Worry | rs1529544 | 3 | 53039455 | A | G | 2.69E-09 | 3p21.1 | *SFMBT1* |
| 256 | UC | Worry | rs34112392 | 5 | 149616316 | C | T | 5.07E-09 | 5q32 | *CAMK2A* |
| 257 | UC | Worry | rs6556416 | 5 | 158818745 | A | C | 2.07E-08 | 5q33.3 | *IL12B/LINC01845* |
| 258 | UC | Worry | rs6933404 | 6 | 137959235 | C | T | 1.04E-09 | 6q23.3 | *BTF3L4P3/LINC02539* |
| 259 | UC | Worry | rs7776797 | 7 | 107493598 | C | T | 3.17E-14 | 7q31.1 | *PIGCP2/DLD* |
| 260 | UC | Worry | rs10758669 | 9 | 4981602 | A | C | 6.65E-11 | 9p24.1 | *HNRNPA1P41/JAK2* |
| 261 | UC | Worry | rs3812565 | 9 | 139272502 | C | T | 2.75E-12 | 9q34.3 | *SNAPC4* |
| 262 | UC | Worry | rs12764283 | 10 | 35530460 | A | G | 4.86E-08 | 10p11.21 | *CREM* |
| 263 | UC | Worry | rs6584283 | 10 | 101290301 | C | T | 5.01E-12 | 10q24.2 | *LINC01475* |
| 264 | UC | Worry | rs2212434 | 11 | 76281593 | C | T | 7.59E-11 | 11q13.5 | *EMSY/LINC02757* |
| 265 | UC | Worry | rs474427 | 11 | 114443573 | A | C | 3.30E-08 | 11q23.2 | *REXO2/NXPE2P1* |
| 266 | UC | Worry | rs12817473 | 12 | 68497408 | A | G | 4.81E-14 | 12q15 | *IFNG-AS1* |
| 267 | UC | Worry | rs1359946 | 13 | 27536972 | A | G | 2.31E-08 | 13q12.13 | *FGFR1OP2P1/RPS21P8* |
| 268 | UC | Worry | rs59716545 | 17 | 38031857 | G | T | 1.39E-10 | 17q21.1 | *ZPBP2* |
| 269 | UC | Worry | rs6017342 | 20 | 43065028 | A | C | 4.34E-12 | 20q13.12 | *LINC01620* |
| 270 | UC | Worry | rs6089763 | 20 | 62308354 | C | T | 1.64E-09 | 20q13.33 | *RTEL1/RTEL1-TNFRSF6B* |
| 271 | UC | Worry | rs2836882 | 21 | 40466570 | A | G | 2.35E-16 | 21q22.2 | *RPL23AP12* |
| 272 | UC | Worry | rs7282490 | 21 | 45615741 | A | G | 4.68E-09 | 21q22.3 | *GATD3A* |

Abbreviations: No.: the numerical order of pleiotropic genomic risk locus; A1: effect allele; A2: non-effect allele; CHR: chromosome; POS: position; IBD: Inflammatory bowel disease; IBS: Irritable bowel syndrome; PUD: Peptic ulcer disease; GERD: Gastro-oesophageal reflux disease; CD; Crohn's disease; UC: Ulcerative colitis.

**Table S6**: Shared loci identified based on PLACO results using FUMA.

| **No.Genomic locus** | **Trait 1** | **Trait 2** | **Top SNP** | **CHR** | **POS** | **A1** | **A2** | ***p_PLACO_*** | **Region** | **Mapped gene** |
| --- | --- | --- | --- | --- | --- | --- | --- | --- | --- | --- |
| 1 | CD | Depressed | rs4385674 | 1 | 67684934 | C | T | 2.85E-10 | 1p31.3 | *IL23R* |
| 2 | CD | Depressed | rs62192912 | 2 | 234144942 | C | T | 1.76E-11 | 2q37.1 | *ATG16L1* |
| 3 | CD | Depressed | rs1873625 | 3 | 49666964 | A | C | 1.74E-17 | 3p21.31 | *BSN* |
| 4 | CD | Depressed | rs683194 | 3 | 107299698 | A | G | 2.01E-10 | 3q13.12 | *BBX* |
| 5 | CD | Depressed | rs13135092 | 4 | 103198082 | A | G | 5.26E-09 | 4q24 | *SLC39A8* |
| 6 | CD | Depressed | rs444210 | 6 | 167390242 | A | G | 1.46E-10 | 6q27 | *RNASET2/MIR3939* |
| 7 | CD | Depressed | rs72796311 | 16 | 50626821 | A | G | 5.04E-13 | 16q12.1 | *NKD1* |
| 8 | CD | Depressed | rs113589236 | 17 | 43981795 | A | G | 2.06E-14 | 17q21.31 | *ARHGAP27* |
| 9 | CD | Depressed | rs12963463 | 18 | 53099093 | C | T | 1.27E-08 | 18q21.2 | *TCF4* |
| 10 | CD | Depressed | rs6032660 | 20 | 44730245 | A | G | 1.14E-08 | 20q13.12 | *RPL13P2/CD40* |
| 11 | CD | Depressed | rs41177 | 22 | 30438447 | A | G | 1.53E-08 | 22q12.2 | *HORMAD2-AS1* |
| 12 | GERD | Depressed | rs80245686 | 1 | 174750751 | G | T | 1.35E-08 | 1q25.1 | *RABGAP1L* |
| 13 | GERD | Depressed | rs4260227 | 2 | 67843537 | A | G | 4.16E-08 | 2p14 | *LINC02831* |
| 14 | GERD | Depressed | rs12634780 | 3 | 50043341 | A | T | 2.69E-10 | 3p21.31 | *ACTL11P/MST1R* |
| 15 | GERD | Depressed | rs41286287 | 6 | 29579991 | A | T | 1.75E-10 | 6p22.1 | *GABBR1* |
| 16 | GERD | Depressed | rs10953620 | 7 | 109173373 | A | C | 6.81E-09 | 7q31.1 | *BUB3P1/EIF3IP1* |
| 17 | GERD | Depressed | rs990153 | 7 | 126202904 | C | T | 3.09E-08 | 7q31.33 | *GRM8* |
| 18 | GERD | Depressed | rs3793577 | 9 | 23737627 | A | G | 1.19E-08 | 9p21.3 | *ELAVL2* |
| 19 | GERD | Depressed | rs1055447 | 11 | 47186424 | A | C | 2.41E-08 | 11p11.2 | *ARFGAP2* |
| 20 | GERD | Depressed | rs2186709 | 11 | 112843616 | A | G | 5.49E-12 | 11q23.2 | *LINC02763/NCAM1* |
| 21 | GERD | Depressed | rs4245147 | 11 | 113318007 | C | T | 2.94E-08 | 11q23.2 | *DRD2* |
| 22 | GERD | Depressed | rs9571745 | 13 | 67843475 | A | C | 3.85E-08 | 13q21.32 | *LOC105370246* |
| 23 | GERD | Depressed | rs4483743 | 13 | 68195416 | A | G | 3.54E-08 | 13q21.32 | *LOC105370249* |
| 24 | GERD | Depressed | rs2193596 | 14 | 75234101 | G | T | 2.62E-09 | 14q24.3 | *YLPM1* |
| 25 | GERD | Depressed | rs9915022 | 17 | 11487165 | A | C | 4.32E-08 | 17p12 | *SHISA6* |
| 26 | GERD | Depressed | rs2732686 | 17 | 44338735 | C | T | 6.91E-17 | 17q21.31 | *ARL17B* |
| 27 | GERD | Depressed | rs11665070 | 18 | 35152563 | A | G | 4.49E-10 | 18q12.2 | *CELF4/MIR4318* |
| 28 | GERD | Depressed | rs784256 | 18 | 53398626 | A | G | 3.93E-08 | 18q21.2 | *TCF4/LINC01415* |
| 29 | IBD | Depressed | rs998884 | 2 | 148539852 | A | G | 3.35E-08 | 2q22.3 | *RNA5SP106/ACVR2A* |
| 30 | IBD | Depressed | rs6741429 | 2 | 199545315 | A | T | 8.72E-09 | 2q33.1 | *PLCL1* |
| 31 | IBD | Depressed | rs4625 | 3 | 49572140 | A | G | 3.01E-13 | 3p21.31 | *DAG1* |
| 32 | IBS | Depressed | rs301806 | 1 | 8482078 | C | T | 1.87E-09 | 1p36.23 | *RERE* |
| 33 | IBS | Depressed | rs1452120 | 3 | 85222123 | C | T | 1.89E-08 | 3p12.1 | *CADM2* |
| 34 | IBS | Depressed | rs1280624 | 3 | 136496690 | C | T | 1.21E-08 | 3q22.3 | *STAG1* |
| 35 | IBS | Depressed | rs3807866 | 7 | 12250378 | A | G | 9.75E-09 | 7p21.3 | *TMEM106B* |
| 36 | IBS | Depressed | rs10757427 | 9 | 23377823 | G | T | 3.81E-08 | 9p21.3 | *LINC01239/SUMO2P2* |
| 37 | IBS | Depressed | rs10821129 | 9 | 96222617 | A | G | 2.80E-09 | 9q22.31 | *FAM120A* |
| 38 | IBS | Depressed | rs1021363 | 10 | 106610839 | A | G | 2.89E-08 | 10q25.1 | *SORCS3* |
| 39 | IBS | Depressed | rs10891490 | 11 | 112885527 | C | T | 9.20E-13 | 11q23.2 | *LINC02763/NCAM1* |
| 40 | IBS | Depressed | rs4309187 | 11 | 113412443 | A | C | 8.94E-09 | 11q23.2 | *DRD2/TMPRSS5* |
| 41 | IBS | Depressed | rs10144845 | 14 | 75237770 | C | T | 2.80E-11 | 14q24.3 | *YLPM1* |
| 42 | IBS | Depressed | rs11665070 | 18 | 35152563 | A | G | 2.44E-11 | 18q12.2 | *CELF4/MIR4318* |
| 43 | IBS | Depressed | rs136402 | 22 | 41598933 | A | G | 1.17E-08 | 22q13.2 | *EP300-AS1/L3MBTL2* |
| 44 | PUD | Depressed | rs4630591 | 17 | 44192568 | C | T | 8.27E-12 | 17q21.31 | *KANSL1* |
| 45 | PUD | Depressed | rs784256 | 18 | 53398626 | A | G | 4.12E-10 | 18q21.2 | *TCF4/LINC01415* |
| 46 | CD | Neuroticism | rs11209008 | 1 | 67623298 | A | G | 6.12E-09 | 1p31.3 | *IL23R* |
| 47 | CD | Neuroticism | rs2488401 | 1 | 197702401 | C | T | 1.84E-09 | 1q31.3 | *DENND1B* |
| 48 | CD | Neuroticism | rs11904361 | 2 | 43848664 | C | T | 3.15E-08 | 2p21 | *PLEKHH2* |
| 49 | CD | Neuroticism | rs62192912 | 2 | 234144942 | C | T | 2.49E-11 | 2q37.1 | *ATG16L1* |
| 50 | CD | Neuroticism | rs11711485 | 3 | 49491983 | G | T | 5.78E-10 | 3p21.31 | *NICN1/RNA5SP130* |
| 51 | CD | Neuroticism | rs683194 | 3 | 107299698 | A | G | 3.99E-10 | 3q13.12 | *BBX* |
| 52 | CD | Neuroticism | rs444210 | 6 | 167390242 | A | G | 3.56E-12 | 6q27 | *RNASET2/MIR3939* |
| 53 | CD | Neuroticism | rs80182618 | 10 | 35496473 | A | G | 4.34E-08 | 10p11.21 | *CREM* |
| 54 | CD | Neuroticism | rs5743292 | 16 | 50759731 | A | T | 8.01E-13 | 16q12.1 | *NOD2* |
| 55 | CD | Neuroticism | rs113589236 | 17 | 43981795 | A | G | 8.45E-17 | 17q21.31 | *MAPT* |
| 56 | CD | Neuroticism | rs12958048 | 18 | 53101598 | A | G | 6.77E-09 | 18q21.2 | *TCF4* |
| 57 | CD | Neuroticism | rs1569723 | 20 | 44742064 | A | C | 4.68E-08 | 20q13.12 | *RPL13P2/CD40* |
| 58 | CD | Neuroticism | rs9614090 | 22 | 30269907 | A | T | 1.88E-10 | 22q12.2 | *ASCC2/MTMR3* |
| 59 | CD | Neuroticism | rs9611555 | 22 | 41713111 | A | G | 1.01E-08 | 22q13.2 | *ZC3H7B* |
| 60 | GERD | Neuroticism | rs219006 | 1 | 37150043 | G | T | 9.95E-09 | 1p34.3 | *FTLP18/GRIK3* |
| 61 | GERD | Neuroticism | rs10912782 | 1 | 174387896 | G | T | 1.25E-08 | 1q25.1 | *RABGAP1L* |
| 62 | GERD | Neuroticism | rs4671537 | 2 | 64146534 | G | T | 4.55E-10 | 2p15 | *WDPCP* |
| 63 | GERD | Neuroticism | rs10928377 | 2 | 148900148 | C | T | 4.90E-09 | 2q23.1 | *MBD5* |
| 64 | GERD | Neuroticism | rs7752448 | 6 | 28301099 | A | G | 4.71E-13 | 6p22.1 | *ZSCAN31* |
| 65 | GERD | Neuroticism | rs28459372 | 7 | 126395673 | A | T | 1.01E-08 | 7q31.33 | *GRM8* |
| 66 | GERD | Neuroticism | rs10119773 | 9 | 23736400 | A | G | 1.64E-09 | 9p21.3 | *ELAVL2* |
| 67 | GERD | Neuroticism | rs34796300 | 11 | 13315205 | C | T | 1.68E-09 | 11p15.3 | *ARNTL* |
| 68 | GERD | Neuroticism | rs2186874 | 11 | 112912724 | C | T | 5.20E-14 | 11q23.2 | *NCAM1* |
| 69 | GERD | Neuroticism | rs2471855 | 11 | 113299500 | C | G | 1.77E-08 | 11q23.2 | *DRD2* |
| 70 | GERD | Neuroticism | rs9571745 | 13 | 67843475 | A | C | 3.12E-08 | 13q21.32 | *LOC105370246* |
| 71 | GERD | Neuroticism | rs2193596 | 14 | 75234101 | G | T | 1.64E-09 | 14q24.3 | *YLPM1* |
| 72 | GERD | Neuroticism | rs2732686 | 17 | 44338735 | C | T | 8.28E-18 | 17q21.31 | *MAPK8IP1P1/ARL17B* |
| 73 | GERD | Neuroticism | rs11665070 | 18 | 35152563 | A | G | 3.65E-11 | 18q12.2 | *CELF4/MIR4318* |
| 74 | GERD | Neuroticism | rs9611555 | 22 | 41713111 | A | G | 2.80E-08 | 22q13.2 | *ZC3H7B* |
| 75 | IBD | Neuroticism | rs998884 | 2 | 148539852 | A | G | 6.08E-10 | 2q22.3 | *RNA5SP106/ACVR2A* |
| 76 | IBD | Neuroticism | rs483143 | 6 | 27846744 | C | G | 1.77E-08 | 6p22.1 | *H4C13/H3C12* |
| 77 | IBS | Neuroticism | rs1729951 | 3 | 136500733 | G | T | 2.02E-11 | 3q22.3 | *PPP2R3A* |
| 78 | IBS | Neuroticism | rs2194027 | 5 | 87822672 | A | T | 2.96E-09 | 5q14.3 | *LINC00461* |
| 79 | IBS | Neuroticism | rs3807866 | 7 | 12250378 | A | G | 1.99E-08 | 7p21.3 | *TMEM106B* |
| 80 | IBS | Neuroticism | rs7869969 | 9 | 96217447 | A | G | 6.00E-11 | 9q22.31 | *FAM120A* |
| 81 | IBS | Neuroticism | rs10891490 | 11 | 112885527 | C | T | 6.32E-15 | 11q23.2 | *NCAM1* |
| 82 | IBS | Neuroticism | rs4938023 | 11 | 113374847 | G | T | 3.00E-09 | 11q23.2 | *DRD2/TMPRSS5* |
| 83 | IBS | Neuroticism | rs55707505 | 14 | 75362552 | C | T | 2.63E-11 | 14q24.3 | *DLST* |
| 84 | IBS | Neuroticism | rs11665070 | 18 | 35152563 | A | G | 8.93E-13 | 18q12.2 | *CELF4/MIR4318* |
| 85 | IBS | Neuroticism | rs5758268 | 22 | 41622419 | A | T | 1.05E-13 | 22q13.2 | *L3MBTL2-AS1/L3MBTL2* |
| 86 | PUD | Neuroticism | rs286799 | 5 | 107481865 | A | G | 2.26E-08 | 5q21.3 | *FBXL17* |
| 87 | PUD | Neuroticism | rs608293 | 11 | 31808280 | C | T | 1.04E-08 | 11p13 | *PAX6* |
| 88 | PUD | Neuroticism | rs7948789 | 11 | 112839532 | A | G | 8.07E-09 | 11q23.2 | *NCAM1* |
| 89 | PUD | Neuroticism | rs62063281 | 17 | 44038785 | A | G | 5.16E-12 | 17q21.31 | *MAPT* |
| 90 | UC | Neuroticism | rs10182512 | 2 | 61189469 | A | G | 2.48E-09 | 2p16.1 | *PUS10* |
| 91 | UC | Neuroticism | rs1873625 | 3 | 49666964 | A | C | 3.64E-10 | 3p21.31 | *BSN* |
| 92 | UC | Neuroticism | rs1529544 | 3 | 53039455 | A | G | 1.14E-08 | 3p21.1 | *SFMBT1* |
| 93 | UC | Neuroticism | rs2384288 | 10 | 35343497 | C | T | 4.11E-08 | 10p11.21 | *CUL2* |
| 94 | UC | Neuroticism | rs12817473 | 12 | 68497408 | A | G | 5.17E-10 | 12q15 | *IFNG-AS1* |
| 95 | UC | Neuroticism | rs34003767 | 17 | 38194296 | A | G | 9.49E-10 | 17q21.1 | *MED24* |
| 96 | UC | Neuroticism | rs1724390 | 17 | 43663247 | A | C | 1.52E-08 | 17q21.31 | *RPS26P8/LINC02210* |
| 97 | UC | Neuroticism | rs2958162 | 18 | 53083865 | C | T | 4.38E-08 | 18q21.2 | *TCF4* |
| 98 | GERD | Worry | rs12073487 | 1 | 98315061 | A | T | 3.62E-09 | 1p21.3 | *DPYD* |
| 99 | GERD | Worry | rs2694635 | 2 | 61676940 | C | T | 2.76E-08 | 2p15 | *USP34* |
| 100 | GERD | Worry | rs4671537 | 2 | 64146534 | G | T | 1.79E-08 | 2p14 | *VPS54/PELI1* |
| 101 | GERD | Worry | rs352139 | 3 | 52258372 | C | T | 2.14E-09 | 3p21.2 | *ALAS1* |
| 102 | GERD | Worry | rs4713177 | 6 | 28673998 | A | G | 1.79E-13 | 6p22.1 | *GPR89P/RSL24D1P1* |
| 103 | GERD | Worry | rs13262595 | 8 | 143316970 | A | G | 3.37E-09 | 8q24.3 | *TSNARE1* |
| 104 | GERD | Worry | rs11191424 | 10 | 104625886 | A | G | 5.39E-10 | 10q24.32 | *BORCS7-ASMT* |
| 105 | GERD | Worry | rs7105462 | 11 | 112912048 | A | G | 3.93E-12 | 11q23.2 | *NCAM1* |
| 106 | GERD | Worry | rs9571745 | 13 | 67843475 | A | C | 1.38E-08 | 13q21.32 | *LOC105370246* |
| 107 | GERD | Worry | rs7160852 | 14 | 75239424 | C | T | 2.88E-09 | 14q24.3 | *YLPM1* |
| 108 | GERD | Worry | rs7207400 | 17 | 43824360 | C | T | 8.00E-14 | 17q21.31 | *RPS26P8/LINC02210* |
| 109 | GERD | Worry | rs11665070 | 18 | 35152563 | A | G | 4.22E-09 | 18q12.2 | *CELF4/MIR4318* |
| 110 | IBS | Worry | rs11800001 | 1 | 153870850 | C | T | 9.54E-09 | 1q21.3 | *GATAD2B* |
| 111 | IBS | Worry | rs6738494 | 2 | 200697058 | A | C | 1.34E-08 | 2q33.1 | *FTCDNL1* |
| 112 | IBS | Worry | rs352140 | 3 | 52256697 | C | T | 3.32E-09 | 3p21.2 | *TLR9* |
| 113 | IBS | Worry | rs6774985 | 3 | 85513000 | C | T | 7.12E-13 | 3p12.1 | *CADM2* |
| 114 | IBS | Worry | rs1280624 | 3 | 136496690 | C | T | 1.62E-12 | 3q22.3 | *STAG1* |
| 115 | IBS | Worry | rs2195613 | 5 | 87644310 | A | G | 2.75E-10 | 5q14.3 | *TMEM161B-AS1* |
| 116 | IBS | Worry | rs10992744 | 9 | 96230194 | A | G | 3.34E-09 | 9q22.31 | *FAM120A* |
| 117 | IBS | Worry | rs7947502 | 11 | 112909396 | C | T | 4.83E-14 | 11q23.2 | *NCAM1* |
| 118 | IBS | Worry | rs9527336 | 13 | 55783042 | A | G | 1.31E-08 | 13q21.1 | *MIR5007* |
| 119 | IBS | Worry | rs10144845 | 14 | 75237770 | C | T | 2.17E-11 | 14q24.3 | *YLPM1* |
| 120 | IBS | Worry | rs11665070 | 18 | 35152563 | A | G | 1.38E-10 | 18q12.2 | *CELF4/MIR4318* |
| 121 | IBS | Worry | rs136402 | 22 | 41598933 | A | G | 2.00E-11 | 22q13.2 | *EP300-AS1/L3MBTL2* |
| 122 | PUD | Worry | rs79205304 | 2 | 164600989 | A | C | 2.06E-08 | 2q24.3 | *FIGN* |
| 123 | PUD | Worry | rs1248857 | 3 | 85018612 | A | G | 2.41E-09 | 3p12.1 | *CADM2* |
| 124 | PUD | Worry | rs3026401 | 11 | 31807524 | C | T | 3.13E-09 | 11p13 | *ELP4/PAX6* |
| 125 | PUD | Worry | rs62063281 | 17 | 44038785 | A | G | 4.49E-09 | 17q21.31 | *MAPT* |
| 126 | UC | Worry | rs4654925 | 1 | 20227723 | C | G | 1.06E-09 | 1p36.13 | *OTUD3* |
| 127 | UC | Worry | rs6685659 | 1 | 227089930 | C | G | 4.60E-08 | 1q42.13 | *PSEN2* |
| 128 | UC | Worry | rs10182512 | 2 | 61189469 | A | G | 5.55E-10 | 2p16.1 | *PUS10* |
| 129 | UC | Worry | rs56322003 | 2 | 199735083 | C | T | 2.98E-11 | 2q33.1 | *NA* |
| 130 | UC | Worry | rs1529544 | 3 | 53039455 | A | G | 6.86E-10 | 3p21.1 | *SFMBT1* |
| 131 | UC | Worry | rs7611991 | 3 | 85759558 | A | G | 5.97E-09 | 3p12.1 | *CADM2* |
| 132 | UC | Worry | rs34003767 | 17 | 38194296 | A | G | 4.10E-08 | 17q21.1 | *MED24* |

Abbreviations: No.: the numerical order of pleiotropic genomic risk locus; A1: effect allele; A2: non-effect allele; CHR: chromosome; POS: position; IBD: Inflammatory bowel disease; IBS: Irritable bowel syndrome; PUD: Peptic ulcer disease; GERD: Gastro-oesophageal reflux disease; CD; Crohn's disease; UC: Ulcerative colitis.

**Table S7**: The shared loci identified by comparing the FUMA results of MTAG and PLACO.

| **Trait 1** | **Trait 2** | ***p*_MTAG_** | ***p*_PLACO_** | **Region** | **PLACO** | | **MTAG** | |
| --- | --- | --- | --- | --- | --- | --- | --- | --- |
|  |  |  |  |  | **Top SNP** | **Nearest gene** | **Top SNP** | **Nearest gene** |
| CD | Depressed | 4.63E-52 | 2.85E-10 | 1p31.3 | rs4385674 | *C1orf141/IL23R* | rs11209026 | *IL23R* |
| CD | Depressed | 1.90E-08 | 1.76E-11 | 2q37.1 | rs62192912 | *ATG16L1* | rs7423615 | *SP140* |
| CD | Depressed | 2.25E-37 | 1.76E-11 | 2q37.1 | rs62192912 | *ATG16L1* | rs3816234 | *ATG16L1* |
| CD | Depressed | 2.63E-12 | 1.74E-17 | 3p21.31 | rs1873625 | *BSN* | rs13096760 | *NICN1* |
| CD | Depressed | 2.49E-09 | 5.26E-09 | 4q24 | rs13135092 | *SLC39A8* | rs13135092 | *SLC39A8* |
| CD | Depressed | 1.75E-13 | 1.46E-10 | 6q27 | rs444210 | *RP1-167A14.2* | rs444210 | *RP1-167A14.2* |
| CD | Depressed | 1.03E-08 | 5.04E-13 | 16q12.1 | rs72796311 | *NKD1* | rs75294107 | *ZNF423* |
| CD | Depressed | 2.28E-59 | 5.04E-13 | 16q12.1 | rs72796311 | *NKD1* | rs2076756 | *NOD2* |
| GERD | Depressed | 1.23E-09 | 4.16E-08 | 2p14 | rs4260227 | *AC010987.5* | rs1430788 | *AC010987.5* |
| GERD | Depressed | 5.57E-10 | 2.69E-10 | 3p21.31 | rs12634780 | *RBM6* | rs73079003 | *CDHR4* |
| GERD | Depressed | 3.39E-13 | 1.75E-10 | 6p22.1 | rs41286287 | *GABBR1* | rs7752448 | *ZSCAN31* |
| GERD | Depressed | 1.65E-11 | 5.49E-12 | 11q23.2 | rs2186709 | *NCAM1* | rs1940722 | *NCAM1* |
| GERD | Depressed | 1.65E-11 | 2.94E-08 | 11q23.2 | rs4245147 | *DRD2* | rs1940722 | *NCAM1* |
| GERD | Depressed | 2.09E-08 | 4.32E-08 | 17p12 | rs9915022 | *DNAH9* | rs11652896 | *SHISA6* |
| IBS | Depressed | 8.94E-09 | 1.87E-09 | 1p36.23 | rs301806 | *RERE* | rs301806 | *RERE* |
| IBS | Depressed | 2.31E-08 | 1.21E-08 | 3q22.3 | rs1280624 | *RP11-102M11.2* | rs1609391 | *NCK1* |
| IBS | Depressed | 1.78E-09 | 9.75E-09 | 7p21.3 | rs3807866 | *TMEM106B* | rs3807866 | *TMEM106B* |
| IBS | Depressed | 5.23E-10 | 2.80E-09 | 9q22.31 | rs10821129 | *FAM120A* | rs10821129 | *FAM120A* |
| IBS | Depressed | 3.47E-12 | 9.20E-13 | 11q23.2 | rs10891490 | *NCAM1* | rs10891490 | *NCAM1* |
| IBS | Depressed | 3.47E-12 | 8.94E-09 | 11q23.2 | rs4309187 | *DRD2* | rs10891490 | *NCAM1* |
| IBS | Depressed | 7.48E-09 | 9.20E-13 | 11q23.2 | rs10891490 | *NCAM1* | rs4245154 | *DRD2* |
| IBS | Depressed | 7.48E-09 | 8.94E-09 | 11q23.2 | rs4309187 | *DRD2* | rs4245154 | *DRD2* |
| IBS | Depressed | 2.03E-10 | 2.80E-11 | 14q24.3 | rs10144845 | *YLPM1* | rs10144845 | *YLPM1* |
| IBS | Depressed | 1.52E-10 | 2.44E-11 | 18q12.2 | rs11665070 | *CELF4* | rs11665070 | *CELF4* |
| IBS | Depressed | 1.45E-09 | 1.17E-08 | 22q13.2 | rs136402 | *L3MBTL2* | rs136402 | *L3MBTL2* |
| PUD | Depressed | 4.27E-09 | 4.12E-10 | 18q21.2 | rs784256 | *RP11-397A16.1* | rs613872 | *TCF4* |
| CD | Neuroticism | 3.35E-52 | 6.12E-09 | 1p31.3 | rs11209008 | *C1orf141* | rs11209026 | *IL23R* |
| CD | Neuroticism | 9.05E-10 | 3.15E-08 | 2p21 | rs11904361 | *Y_RNA* | rs10186441 | *Y_RNA* |
| CD | Neuroticism | 1.67E-08 | 2.49E-11 | 2q37.1 | rs62192912 | *ATG16L1* | rs7423615 | *SP140* |
| CD | Neuroticism | 9.40E-38 | 2.49E-11 | 2q37.1 | rs62192912 | *ATG16L1* | rs3816234 | *ATG16L1* |
| CD | Neuroticism | 1.34E-12 | 5.78E-10 | 3p21.31 | rs11711485 | *RNA5SP130* | rs13096760 | *NICN1* |
| CD | Neuroticism | 7.21E-14 | 3.56E-12 | 6q27 | rs444210 | *RP1-167A14.2* | rs444210 | *RP1-167A14.2* |
| CD | Neuroticism | 2.52E-10 | 4.34E-08 | 10p11.21 | rs80182618 | *CREM* | rs2505640 | *CREM* |
| CD | Neuroticism | 1.26E-08 | 8.01E-13 | 16q12.1 | rs5743292 | *NOD2* | rs75294107 | *ZNF423* |
| CD | Neuroticism | 1.37E-59 | 8.01E-13 | 16q12.1 | rs5743292 | *NOD2* | rs2076756 | *NOD2* |
| GERD | Neuroticism | 1.80E-09 | 4.55E-10 | 2p15 | rs4671537 | *VPS54* | rs66595136 | *VPS54* |
| GERD | Neuroticism | 2.80E-14 | 4.71E-13 | 6p22.1 | rs7752448 | *ZSCAN31* | rs7752448 | *ZSCAN31* |
| GERD | Neuroticism | 1.53E-11 | 5.20E-14 | 11q23.2 | rs2186874 | *NCAM1* | rs1940722 | *NCAM1* |
| GERD | Neuroticism | 1.53E-11 | 1.77E-08 | 11q23.2 | rs2471855 | *DRD2* | rs1940722 | *NCAM1* |
| GERD | Neuroticism | 4.94E-08 | 3.65E-11 | 18q12.2 | rs11665070 | *CELF4* | rs11665070 | *CELF4* |
| IBS | Neuroticism | 1.73E-11 | 2.02E-11 | 3q22.3 | rs1729951 | *RP11-102M11.2* | rs1729951 | *RP11-102M11.2* |
| IBS | Neuroticism | 2.97E-09 | 2.96E-09 | 5q14.3 | rs2194027 | *LINC00461* | rs7706932 | *CTC-498M16.4* |
| IBS | Neuroticism | 8.19E-09 | 1.99E-08 | 7p21.3 | rs3807866 | *TMEM106B* | rs3807866 | *TMEM106B* |
| IBS | Neuroticism | 6.33E-11 | 6.00E-11 | 9q22.31 | rs7869969 | *FAM120A* | rs10821129 | *FAM120A* |
| IBS | Neuroticism | 8.34E-15 | 6.32E-15 | 11q23.2 | rs10891490 | *NCAM1* | rs7105462 | *NCAM1* |
| IBS | Neuroticism | 8.34E-15 | 3.00E-09 | 11q23.2 | rs4938023 | *DRD2* | rs7105462 | *NCAM1* |
| IBS | Neuroticism | 9.00E-11 | 6.32E-15 | 11q23.2 | rs10891490 | *NCAM1* | rs7111031 | *DRD2* |
| IBS | Neuroticism | 9.00E-11 | 3.00E-09 | 11q23.2 | rs4938023 | *DRD2* | rs7111031 | *DRD2* |
| IBS | Neuroticism | 9.86E-12 | 2.63E-11 | 14q24.3 | rs55707505 | *DLST* | rs10144845 | *YLPM1* |
| IBS | Neuroticism | 2.17E-14 | 8.93E-13 | 18q12.2 | rs11665070 | *CELF4* | rs11665070 | *CELF4* |
| IBS | Neuroticism | 1.19E-13 | 1.05E-13 | 22q13.2 | rs5758268 | *L3MBTL2* | rs3818003 | *L3MBTL2/RP4-756G23.5* |
| UC | Neuroticism | 8.80E-14 | 2.48E-09 | 2p16.1 | rs10182512 | *PUS10* | rs10182512 | *PUS10* |
| UC | Neuroticism | 1.50E-12 | 3.64E-10 | 3p21.31 | rs1873625 | *BSN* | rs1131095 | *APEH* |
| UC | Neuroticism | 3.94E-08 | 1.14E-08 | 3p21.1 | rs1529544 | *SFMBT1/RP11-894J14.5* | rs1529544 | *SFMBT1/RP11-894J14.5* |
| UC | Neuroticism | 4.03E-15 | 5.17E-10 | 12q15 | rs12817473 | *IFNG-AS1* | rs12817473 | *IFNG-AS1* |
| UC | Neuroticism | 1.44E-10 | 9.49E-10 | 17q21.1 | rs34003767 | *MED24* | rs59716545 | *ZPBP2* |
| GERD | Worry | 9.88E-09 | 2.76E-08 | 2p15 | rs2694635 | *USP34* | rs66595136 | *VPS54* |
| GERD | Worry | 6.40E-14 | 1.79E-13 | 6p22.1 | rs4713177 | *RPSAP2* | rs7752448 | *ZSCAN31* |
| GERD | Worry | 1.05E-09 | 3.93E-12 | 11q23.2 | rs7105462 | *NCAM1* | rs2186874 | *NCAM1* |
| IBS | Worry | 1.55E-08 | 9.54E-09 | 1q21.3 | rs11800001 | *GATAD2B* | rs11800001 | *GATAD2B* |
| IBS | Worry | 2.41E-08 | 1.34E-08 | 2q33.1 | rs6738494 | *FTCDNL1* | rs6738494 | *FTCDNL1* |
| IBS | Worry | 4.71E-09 | 3.32E-09 | 3p21.2 | rs352140 | *TLR9* | rs352140 | *TLR9:TLR9* |
| IBS | Worry | 3.92E-13 | 7.12E-13 | 3p12.1 | rs6774985 | *CADM2* | rs62250713 | *CADM2* |
| IBS | Worry | 9.71E-12 | 1.62E-12 | 3q22.3 | rs1280624 | *RP11-102M11.2* | rs1280624 | *RP11-102M11.2* |
| IBS | Worry | 3.57E-10 | 2.75E-10 | 5q14.3 | rs2195613 | *TMEM161B-AS1* | rs7706932 | *CTC-498M16.4* |
| IBS | Worry | 4.09E-10 | 3.34E-09 | 9q22.31 | rs10992744 | *FAM120A* | rs10992744 | *FAM120A* |
| IBS | Worry | 3.21E-13 | 4.83E-14 | 11q23.2 | rs7947502 | *NCAM1* | rs7947502 | *NCAM1* |
| IBS | Worry | 3.59E-08 | 1.31E-08 | 13q21.1 | rs9527336 | *MIR5007* | rs9527336 | *MIR5007* |
| IBS | Worry | 2.07E-11 | 2.17E-11 | 14q24.3 | rs10144845 | *YLPM1* | rs55707505 | *DLST* |
| IBS | Worry | 4.83E-10 | 1.38E-10 | 18q12.2 | rs11665070 | *CELF4* | rs11665070 | *CELF4* |
| IBS | Worry | 2.93E-12 | 2.00E-11 | 22q13.2 | rs136402 | *L3MBTL2* | rs136402 | *L3MBTL2* |
| PUD | Worry | 2.18E-08 | 2.06E-08 | 2q24.3 | rs79205304 | *AC092684.1* | rs79205304 | *AC092684.1* |
| UC | Worry | 1.70E-24 | 1.06E-09 | 1p36.13 | rs4654925 | *OTUD3* | rs10737481 | *RP11-91K11.2* |
| UC | Worry | 3.60E-14 | 5.55E-10 | 2p16.1 | rs10182512 | *PUS10* | rs10182512 | *PUS10* |
| UC | Worry | 3.24E-10 | 2.98E-11 | 2q33.1 | rs56322003 | *AC018717.1* | rs62180151 | *AC018717.1* |
| UC | Worry | 2.69E-09 | 6.86E-10 | 3p21.1 | rs1529544 | *SFMBT1/RP11-894J14.5* | rs1529544 | *SFMBT1/RP11-894J14.5* |
| UC | Worry | 1.39E-10 | 4.10E-08 | 17q21.1 | rs34003767 | *MED24* | rs59716545 | *ZPBP2* |

Abbreviations: IBD: Inflammatory bowel disease; IBS: Irritable bowel syndrome; PUD: Peptic ulcer disease; GERD: Gastro-oesophageal reflux disease; CD; Crohn's disease; UC: Ulcerative colitis.

**Table S8**: The lead SNPs identified by comparing the FUMA results of MTAG and PLACO.

| **Trait1** | **Trait2** | **Lead SNP** | **CHR** | **POS** | **A1** | **A2** | ***p*_PLACO_** | ***p*_MTAG_** | **Nearest gene** | **Function** | **CADD** | **RDB** |
| --- | --- | --- | --- | --- | --- | --- | --- | --- | --- | --- | --- | --- |
| GERD | Neuroticism | rs11665070 | 18 | 35152563 | A | G | 3.65E-11 | 4.94E-08 | *CELF4* | intergenic | 11.24 | 3a |
| GERD | Worry | rs41291770 | 6 | 29594265 | C | T | 1.87E-08 | 4.35E-08 | *GABBR1* | intronic | 1.67 | 5 |
| UC | Neuroticism | rs1529544 | 3 | 53039455 | A | G | 1.14E-08 | 3.94E-08 | *SFMBT1/RP11-894J14.5* | intronic | 1.972 | NA |
| IBS | Worry | rs9527336 | 13 | 55783042 | A | G | 1.31E-08 | 3.59E-08 | *MIR5007* | intergenic | 12.1 | 7 |
| IBS | Worry | rs6738494 | 2 | 200697058 | A | C | 1.34E-08 | 2.41E-08 | *FTCDNL1* | intronic | 1.287 | 7 |
| PUD | Worry | rs79205304 | 2 | 164600989 | A | C | 2.06E-08 | 2.18E-08 | *AC092684.1* | intergenic | 3.397 | 6 |
| IBS | Worry | rs11800001 | 1 | 153870850 | C | T | 9.54E-09 | 1.55E-08 | *GATAD2B* | intronic | 0.674 | 6 |
| IBS | Neuroticism | rs17570807 | 18 | 35242636 | A | G | 2.89E-08 | 9.28E-09 | *MIR4318* | intergenic | 2.003 | 5 |
| IBS | Depressed | rs301806 | 1 | 8482078 | C | T | 1.87E-09 | 8.94E-09 | *RERE* | intronic | 0.117 | 4 |
| IBS | Neuroticism | rs3807866 | 7 | 12250378 | A | G | 1.99E-08 | 8.19E-09 | *TMEM106B* | upstream | 7.544 | NA |
| IBS | Worry | rs352140 | 3 | 52256697 | C | T | 3.32E-09 | 4.71E-09 | *TLR9* | exonic | 0.061 | NA |
| UC | Worry | rs1529544 | 3 | 53039455 | A | G | 6.86E-10 | 2.69E-09 | *SFMBT1/RP11-894J14.5* | intronic | 1.972 | NA |
| CD | Depressed | rs13135092 | 4 | 103198082 | A | G | 5.26E-09 | 2.49E-09 | *SLC39A8* | intronic | 10.31 | 6 |
| GERD | Neuroticism | rs41291770 | 6 | 29594265 | C | T | 3.85E-11 | 2.35E-09 | *GABBR1* | intronic | 1.67 | 5 |
| IBS | Depressed | rs3807866 | 7 | 12250378 | A | G | 9.75E-09 | 1.78E-09 | *TMEM106B* | upstream | 7.544 | NA |
| IBS | Depressed | rs136402 | 22 | 41598933 | A | G | 1.17E-08 | 1.45E-09 | *L3MBTL2* | intergenic | 4.12 | 7 |
| GERD | Neuroticism | rs55706012 | 6 | 26266311 | A | C | 1.45E-09 | 1.18E-09 | *HIST1H3G* | intergenic | 2.341 | 7 |
| IBS | Depressed | rs10821129 | 9 | 96222617 | A | G | 2.80E-09 | 5.23E-10 | *FAM120A* | intronic | 0.079 | 6 |
| IBS | Worry | rs11665070 | 18 | 35152563 | A | G | 1.38E-10 | 4.83E-10 | *CELF4* | intergenic | 11.24 | 3a |
| IBS | Worry | rs10992744 | 9 | 96230194 | A | G | 3.34E-09 | 4.09E-10 | *FAM120A* | intronic | 0.099 | 6 |
| GERD | Depressed | rs9358901 | 6 | 26024436 | G | T | 6.22E-10 | 2.05E-10 | *HIST1H4A* | intergenic | 0.115 | 7 |
| IBS | Depressed | rs10144845 | 14 | 75237770 | C | T | 2.80E-11 | 2.03E-10 | *YLPM1* | intronic | 1.624 | 5 |
| GERD | Neuroticism | rs9358901 | 6 | 26024436 | G | T | 1.36E-09 | 1.64E-10 | *HIST1H4A* | intergenic | 0.115 | 7 |
| IBS | Depressed | rs11665070 | 18 | 35152563 | A | G | 2.44E-11 | 1.52E-10 | *CELF4* | intergenic | 11.24 | 3a |
| CD | Depressed | rs4655709 | 1 | 67903316 | A | G | 5.03E-09 | 3.72E-11 | *SERBP1* | intergenic | 0.416 | 7 |
| IBS | Neuroticism | rs1729951 | 3 | 136500733 | G | T | 2.02E-11 | 1.73E-11 | *RP11-102M11.2* | intergenic | 0.078 | NA |
| IBS | Worry | rs1280624 | 3 | 136496690 | C | T | 1.62E-12 | 9.71E-12 | *RP11-102M11.2* | intergenic | 3.6 | NA |
| IBS | Depressed | rs10891490 | 11 | 112885527 | C | T | 9.20E-13 | 3.47E-12 | *NCAM1* | intronic | 5.228 | 4 |
| IBS | Worry | rs136402 | 22 | 41598933 | A | G | 2.00E-11 | 2.93E-12 | *L3MBTL2* | intergenic | 4.12 | 7 |
| GERD | Depressed | rs7755997 | 6 | 26483048 | A | G | 6.36E-09 | 9.07E-13 | *BTN2A1* | intergenic | 1.687 | 7 |
| GERD | Depressed | rs7752448 | 6 | 28301099 | A | G | 2.64E-09 | 3.39E-13 | *ZSCAN31* | intronic | 5.034 | 4 |
| IBS | Worry | rs7947502 | 11 | 112909396 | C | T | 4.83E-14 | 3.21E-13 | *NCAM1* | intronic | 1.529 | 7 |
| CD | Depressed | rs444210 | 6 | 167390242 | A | G | 1.46E-10 | 1.75E-13 | *RP1-167A14.2* | ncRNA_intronic | 2.788 | NA |
| GERD | Worry | rs13212562 | 6 | 27300310 | A | G | 5.02E-13 | 1.23E-13 | *VN1R10P* | intergenic | 2.083 | 4 |
| GERD | Neuroticism | rs13212562 | 6 | 27300310 | A | G | 6.60E-13 | 1.23E-13 | *VN1R10P* | intergenic | 2.083 | 4 |
| GERD | Worry | rs7755997 | 6 | 26483048 | A | G | 2.26E-10 | 1.22E-13 | *BTN2A1* | intergenic | 1.687 | 7 |
| UC | Neuroticism | rs10182512 | 2 | 61189469 | A | G | 2.48E-09 | 8.80E-14 | *PUS10* | intronic | 1.614 | 6 |
| CD | Neuroticism | rs444210 | 6 | 167390242 | A | G | 3.56E-12 | 7.21E-14 | *RP1-167A14.2* | ncRNA_intronic | 2.788 | NA |
| UC | Worry | rs10182512 | 2 | 61189469 | A | G | 5.55E-10 | 3.60E-14 | *PUS10* | intronic | 1.614 | 6 |
| GERD | Neuroticism | rs7752448 | 6 | 28301099 | A | G | 4.71E-13 | 2.80E-14 | *ZSCAN31* | intronic | 5.034 | 4 |
| IBS | Neuroticism | rs11665070 | 18 | 35152563 | A | G | 8.93E-13 | 2.17E-14 | *CELF4* | intergenic | 11.24 | 3a |
| UC | Neuroticism | rs12817473 | 12 | 68497408 | A | G | 5.17E-10 | 4.03E-15 | *IFNG-AS1* | ncRNA_intronic | 3.283 | 7 |
| CD | Depressed | rs79028154 | 16 | 51013434 | C | T | 2.15E-08 | 7.93E-24 | *RP11-883G14.2* | intergenic | 2.974 | 5 |
| CD | Depressed | rs146528649 | 16 | 50660964 | G | T | 8.34E-10 | 4.68E-38 | *NKD1* | intronic | 0.752 | 5 |

Abbreviations: A1: effect allele; A2: non-effect allele; CHR: chromosome; POS: position; CADD: Combined Annotation-Dependent depletion scores; RDB: RegulomeDB scores; IBD: Inflammatory bowel disease; IBS: Irritable bowel syndrome; PUD: Peptic ulcer disease; GERD: Gastro-oesophageal reflux disease; CD; Crohn's disease; UC: Ulcerative colitis.

**Table S9**: Candidate Pleiotropic Genes Identified by MAGMA.

| **Trait 1** | **Trait 2** | **Gene** | **Entrez ID** | **CHR** | **Start** | **End** | **Z(MTAG)** | **Z(PLACO)** | ***p*_MTAG_** | ***p*_PLACO_** |
| --- | --- | --- | --- | --- | --- | --- | --- | --- | --- | --- |
| CD | Depressed | *AMT* | 275 | 3 | 49454211 | 49460111 | 7.01 | 7.18 | 1.20E-12 | 3.43E-13 |
| CD | Depressed | *APEH* | 327 | 3 | 49711427 | 49720936 | 5.69 | 7.31 | 6.24E-09 | 1.35E-13 |
| CD | Depressed | *ATP2A1* | 487 | 16 | 28889192 | 28915830 | 5.71 | 4.60 | 5.70E-09 | 2.07E-06 |
| CD | Depressed | *ATXN2L* | 11273 | 16 | 28834369 | 28848558 | 5.92 | 4.64 | 1.60E-09 | 1.77E-06 |
| CD | Depressed | *BSN* | 8927 | 3 | 49591922 | 49708982 | 6.70 | 7.32 | 1.07E-11 | 1.27E-13 |
| CD | Depressed | *CAMKV* | 79012 | 3 | 49895414 | 49907655 | 4.66 | 6.52 | 1.57E-06 | 3.46E-11 |
| CD | Depressed | *CDHR4* | 389118 | 3 | 49828165 | 49837254 | 5.97 | 5.91 | 1.17E-09 | 1.71E-09 |
| CD | Depressed | *DAG1* | 1605 | 3 | 49506136 | 49573051 | 7.08 | 6.79 | 7.17E-13 | 5.46E-12 |
| CD | Depressed | *GPX1* | 2876 | 3 | 49394609 | 49395791 | 6.14 | 8.18 | 4.16E-10 | 1.37E-16 |
| CD | Depressed | *IL23R* | 149233 | 1 | 67604590 | 67725662 | 9.08 | 5.65 | 5.59E-20 | 7.81E-09 |
| CD | Depressed | *IL27* | 246778 | 16 | 28510683 | 28518155 | 5.72 | 4.73 | 5.37E-09 | 1.11E-06 |
| CD | Depressed | *IP6K1* | 9807 | 3 | 49761728 | 49823973 | 5.37 | 6.47 | 4.01E-08 | 4.93E-11 |
| CD | Depressed | *LST1* | 7940 | 6 | 31553956 | 31556686 | 5.77 | 6.23 | 3.95E-09 | 2.35E-10 |
| CD | Depressed | *MST1* | 4485 | 3 | 49721380 | 49726196 | 6.62 | 7.59 | 1.75E-11 | 1.61E-14 |
| CD | Depressed | *NICN1* | 84276 | 3 | 49459766 | 49466777 | 6.09 | 6.70 | 5.60E-10 | 1.03E-11 |
| CD | Depressed | *NOD2* | 64127 | 16 | 50727507 | 50766990 | 6.11 | 5.29 | 5.00E-10 | 6.27E-08 |
| CD | Depressed | *RHOA* | 387 | 3 | 49396569 | 49449526 | 6.61 | 6.26 | 1.95E-11 | 1.87E-10 |
| CD | Depressed | *RNF123* | 63891 | 3 | 49726950 | 49758962 | 6.14 | 6.99 | 4.07E-10 | 1.33E-12 |
| CD | Depressed | *SGF29* | 112869 | 16 | 28565249 | 28603111 | 5.76 | 4.68 | 4.30E-09 | 1.45E-06 |
| CD | Depressed | *SH2B1* | 25970 | 16 | 28872939 | 28885534 | 6.45 | 4.83 | 5.71E-11 | 6.92E-07 |
| CD | Depressed | *STK19* | 8859 | 6 | 31938952 | 31949223 | 6.46 | 5.28 | 5.08E-11 | 6.61E-08 |
| CD | Depressed | *SULT1A2* | 6799 | 16 | 28603264 | 28611058 | 5.88 | 4.60 | 2.11E-09 | 2.15E-06 |
| CD | Depressed | *TCTA* | 6988 | 3 | 49449639 | 49453909 | 6.77 | 7.33 | 6.40E-12 | 1.16E-13 |
| CD | Depressed | *THADA* | 63892 | 2 | 43457975 | 43823185 | 5.93 | 5.69 | 1.53E-09 | 6.20E-09 |
| CD | Depressed | *TNXB* | 7148 | 6 | 32008932 | 32077151 | 6.00 | 5.46 | 9.73E-10 | 2.35E-08 |
| CD | Depressed | *TRAIP* | 10293 | 3 | 49866028 | 49893992 | 5.87 | 6.34 | 2.22E-09 | 1.18E-10 |
| CD | Depressed | *TUFM* | 7284 | 16 | 28853732 | 28857729 | 5.46 | 4.70 | 2.35E-08 | 1.28E-06 |
| CD | Depressed | *UBA7* | 7318 | 3 | 49842638 | 49851391 | 5.70 | 4.89 | 5.96E-09 | 5.03E-07 |
| CD | Depressed | *USP4* | 7375 | 3 | 49314577 | 49377536 | 5.22 | 4.67 | 9.10E-08 | 1.48E-06 |
| GERD | Depressed | *BSN* | 8927 | 3 | 49591922 | 49708982 | 5.06 | 5.62 | 2.13E-07 | 9.75E-09 |
| GERD | Depressed | *BTN2A1* | 11120 | 6 | 26458132 | 26476849 | 6.94 | 5.28 | 1.90E-12 | 6.53E-08 |
| GERD | Depressed | *CAMKV* | 79012 | 3 | 49895414 | 49907655 | 5.87 | 6.17 | 2.14E-09 | 3.39E-10 |
| GERD | Depressed | *CDHR4* | 389118 | 3 | 49828165 | 49837254 | 5.22 | 5.25 | 8.92E-08 | 7.80E-08 |
| GERD | Depressed | *DALRD3* | 55152 | 3 | 49052832 | 49058504 | 5.00 | 5.26 | 2.85E-07 | 7.22E-08 |
| GERD | Depressed | *DRD2* | 1813 | 11 | 113280317 | 113346413 | 5.11 | 5.70 | 1.61E-07 | 6.08E-09 |
| GERD | Depressed | *GNAT1* | 2779 | 3 | 50229043 | 50235129 | 4.92 | 4.66 | 4.42E-07 | 1.55E-06 |
| GERD | Depressed | *H1-5* | 3009 | 6 | 27834570 | 27835359 | 4.96 | 4.72 | 3.47E-07 | 1.16E-06 |
| GERD | Depressed | *H1-6* | 3010 | 6 | 26107640 | 26108364 | 5.11 | 5.03 | 1.60E-07 | 2.39E-07 |
| GERD | Depressed | *H2BC15* | 8341 | 6 | 27805544 | 27821533 | 6.33 | 5.50 | 1.19E-10 | 1.95E-08 |
| GERD | Depressed | *H2BC4* | 8347 | 6 | 26113389 | 26124266 | 4.89 | 4.93 | 5.08E-07 | 4.12E-07 |
| GERD | Depressed | *H3C12* | 8356 | 6 | 27858093 | 27858570 | 6.19 | 5.73 | 3.03E-10 | 5.15E-09 |
| GERD | Depressed | *H3C2* | 8358 | 6 | 26031817 | 26032288 | 5.88 | 5.57 | 2.06E-09 | 1.26E-08 |
| GERD | Depressed | *H3C3* | 8352 | 6 | 26045639 | 26046097 | 6.07 | 4.99 | 6.47E-10 | 3.02E-07 |
| GERD | Depressed | *H4C13* | 8368 | 6 | 27840926 | 27841289 | 6.42 | 5.29 | 6.78E-11 | 6.01E-08 |
| GERD | Depressed | *H4C3* | 8364 | 6 | 26104176 | 26104565 | 5.43 | 5.21 | 2.80E-08 | 9.69E-08 |
| GERD | Depressed | *HLA-B* | 3106 | 6 | 31321649 | 31324989 | 5.39 | 4.84 | 3.60E-08 | 6.52E-07 |
| GERD | Depressed | *IP6K1* | 9807 | 3 | 49761728 | 49823973 | 5.38 | 5.74 | 3.81E-08 | 4.65E-09 |
| GERD | Depressed | *LAMB2* | 3913 | 3 | 49158547 | 49170599 | 6.16 | 6.47 | 3.58E-10 | 5.03E-11 |
| GERD | Depressed | *MON1A* | 84315 | 3 | 49946302 | 49967445 | 5.64 | 5.94 | 8.46E-09 | 1.41E-09 |
| GERD | Depressed | *MST1* | 4485 | 3 | 49721380 | 49726196 | 5.83 | 6.18 | 2.85E-09 | 3.26E-10 |
| GERD | Depressed | *MST1R* | 4486 | 3 | 49924435 | 49941311 | 5.47 | 5.87 | 2.26E-08 | 2.21E-09 |
| GERD | Depressed | *MYLPF* | 29895 | 16 | 30386123 | 30389310 | 4.75 | 4.96 | 1.03E-06 | 3.59E-07 |
| GERD | Depressed | *NCAM1* | 4684 | 11 | 112831969 | 113149158 | 5.97 | 5.73 | 1.22E-09 | 5.15E-09 |
| GERD | Depressed | *NICN1* | 84276 | 3 | 49459766 | 49466777 | 5.07 | 5.44 | 1.99E-07 | 2.71E-08 |
| GERD | Depressed | *OR2B2* | 81697 | 6 | 27878963 | 27880174 | 5.31 | 5.13 | 5.49E-08 | 1.45E-07 |
| GERD | Depressed | *PGBD1* | 84547 | 6 | 28249314 | 28270326 | 5.70 | 4.54 | 5.96E-09 | 2.79E-06 |
| GERD | Depressed | *RABGAP1L* | 9910 | 1 | 174128552 | 174964445 | 4.82 | 4.56 | 7.05E-07 | 2.52E-06 |
| GERD | Depressed | *RBM5* | 10181 | 3 | 50126341 | 50156397 | 5.72 | 5.78 | 5.31E-09 | 3.66E-09 |
| GERD | Depressed | *RBM6* | 10180 | 3 | 49977474 | 50114685 | 5.48 | 5.72 | 2.08E-08 | 5.42E-09 |
| GERD | Depressed | *RNF123* | 63891 | 3 | 49726950 | 49758962 | 5.33 | 6.04 | 4.84E-08 | 7.83E-10 |
| GERD | Depressed | *SEMA3F* | 6405 | 3 | 50192562 | 50226508 | 5.55 | 5.56 | 1.41E-08 | 1.32E-08 |
| GERD | Depressed | *TRAIP* | 10293 | 3 | 49866028 | 49893992 | 5.39 | 5.63 | 3.47E-08 | 9.23E-09 |
| GERD | Depressed | *UGP2* | 7360 | 2 | 64068084 | 64118696 | 4.98 | 4.62 | 3.14E-07 | 1.94E-06 |
| IBD | Depressed | *HLA-DQA1* | 3117 | 6 | 32605169 | 32612152 | 5.22 | 5.78 | 8.85E-08 | 3.71E-09 |
| IBD | Depressed | *HLA-DQB1* | 3119 | 6 | 32627241 | 32634466 | 6.38 | 5.58 | 8.58E-11 | 1.23E-08 |
| IBD | Depressed | *HLA-DRB1* | 3123 | 6 | 32546546 | 32557613 | 5.25 | 5.02 | 7.51E-08 | 2.62E-07 |
| IBS | Depressed | *ATF6B* | 1388 | 6 | 32083045 | 32096017 | 4.82 | 4.78 | 7.17E-07 | 8.81E-07 |
| IBS | Depressed | *DLST* | 1743 | 14 | 75348594 | 75370450 | 5.40 | 5.18 | 3.33E-08 | 1.11E-07 |
| IBS | Depressed | *DRD2* | 1813 | 11 | 113280317 | 113346413 | 5.77 | 5.43 | 4.02E-09 | 2.75E-08 |
| IBS | Depressed | *FAM120A* | 23196 | 9 | 96214173 | 96328397 | 5.95 | 5.43 | 1.32E-09 | 2.85E-08 |
| IBS | Depressed | *MPHOSPH9* | 10198 | 12 | 123640943 | 123717785 | 4.95 | 4.81 | 3.71E-07 | 7.68E-07 |
| IBS | Depressed | *NCAM1* | 4684 | 11 | 112831969 | 113149158 | 5.63 | 5.18 | 9.08E-09 | 1.12E-07 |
| IBS | Depressed | *RANGAP1* | 5905 | 22 | 41640781 | 41682255 | 5.34 | 4.77 | 4.59E-08 | 9.14E-07 |
| IBS | Depressed | *RERE* | 473 | 1 | 8412464 | 8877699 | 5.07 | 5.00 | 2.00E-07 | 2.94E-07 |
| IBS | Depressed | *SORCS3* | 22986 | 10 | 106400859 | 107024993 | 5.62 | 4.62 | 9.73E-09 | 1.91E-06 |
| IBS | Depressed | *STAG1* | 10274 | 3 | 136055077 | 136471245 | 5.19 | 4.93 | 1.05E-07 | 4.04E-07 |
| IBS | Depressed | *TMEM106B* | 54664 | 7 | 12250848 | 12276890 | 5.72 | 5.34 | 5.20E-09 | 4.56E-08 |
| IBS | Depressed | *TTC12* | 54970 | 11 | 113185251 | 113244345 | 4.91 | 4.70 | 4.50E-07 | 1.28E-06 |
| IBS | Depressed | *YLPM1* | 56252 | 14 | 75230025 | 75304013 | 5.12 | 5.11 | 1.57E-07 | 1.57E-07 |
| IBS | Depressed | *ZC3H7B* | 23264 | 22 | 41697507 | 41756151 | 5.01 | 4.77 | 2.75E-07 | 9.01E-07 |
| CD | Neuroticism | *AMT* | 275 | 3 | 49454211 | 49460111 | 7.61 | 4.74 | 1.38E-14 | 1.05E-06 |
| CD | Neuroticism | *ATG16L1* | 55054 | 2 | 234160217 | 234204320 | 6.11 | 5.27 | 5.00E-10 | 6.91E-08 |
| CD | Neuroticism | *BSN* | 8927 | 3 | 49591922 | 49708982 | 6.91 | 4.54 | 2.36E-12 | 2.77E-06 |
| CD | Neuroticism | *CAMKV* | 79012 | 3 | 49895414 | 49907655 | 4.86 | 4.67 | 6.02E-07 | 1.48E-06 |
| CD | Neuroticism | *CDHR4* | 389118 | 3 | 49828165 | 49837254 | 6.04 | 4.60 | 7.58E-10 | 2.12E-06 |
| CD | Neuroticism | *CFB* | 629 | 6 | 31913721 | 31919861 | 4.76 | 4.73 | 9.89E-07 | 1.14E-06 |
| CD | Neuroticism | *CREM* | 1390 | 10 | 35415769 | 35501886 | 6.01 | 5.02 | 9.50E-10 | 2.61E-07 |
| CD | Neuroticism | *CUL2* | 8453 | 10 | 35297479 | 35379570 | 5.99 | 5.04 | 1.05E-09 | 2.31E-07 |
| CD | Neuroticism | *CYLD* | 1540 | 16 | 50775961 | 50835847 | 7.15 | 5.62 | 4.37E-13 | 9.63E-09 |
| CD | Neuroticism | *GPX1* | 2876 | 3 | 49394609 | 49395791 | 6.37 | 5.78 | 9.59E-11 | 3.66E-09 |
| CD | Neuroticism | *HLA-B* | 3106 | 6 | 31321649 | 31324989 | 5.39 | 4.96 | 3.49E-08 | 3.45E-07 |
| CD | Neuroticism | *IL23R* | 149233 | 1 | 67604590 | 67725662 | 8.30 | 4.89 | 5.35E-17 | 4.93E-07 |
| CD | Neuroticism | *IL27* | 246778 | 16 | 28510683 | 28518155 | 5.68 | 4.54 | 6.73E-09 | 2.75E-06 |
| CD | Neuroticism | *IP6K1* | 9807 | 3 | 49761728 | 49823973 | 5.49 | 5.10 | 2.04E-08 | 1.68E-07 |
| CD | Neuroticism | *LST1* | 7940 | 6 | 31553956 | 31556686 | 5.55 | 6.06 | 1.46E-08 | 6.98E-10 |
| CD | Neuroticism | *MSH5* | 4439 | 6 | 31707725 | 31730455 | 4.82 | 5.21 | 7.17E-07 | 9.36E-08 |
| CD | Neuroticism | *MST1* | 4485 | 3 | 49721380 | 49726196 | 6.81 | 5.37 | 4.76E-12 | 3.96E-08 |
| CD | Neuroticism | *NOD2* | 64127 | 16 | 50727507 | 50766990 | 6.11 | 6.16 | 5.00E-10 | 3.53E-10 |
| CD | Neuroticism | *PUS10* | 150962 | 2 | 61167548 | 61245365 | 5.71 | 4.98 | 5.67E-09 | 3.24E-07 |
| CD | Neuroticism | *RNF123* | 63891 | 3 | 49726950 | 49758962 | 6.28 | 5.29 | 1.67E-10 | 6.17E-08 |
| CD | Neuroticism | *SLC22A4* | 6583 | 5 | 131630145 | 131679899 | 5.96 | 4.63 | 1.29E-09 | 1.85E-06 |
| CD | Neuroticism | *SLC22A5* | 6584 | 5 | 131705396 | 131731306 | 6.53 | 5.00 | 3.40E-11 | 2.85E-07 |
| CD | Neuroticism | *STK19* | 8859 | 6 | 31938952 | 31949223 | 6.37 | 6.81 | 9.46E-11 | 4.77E-12 |
| CD | Neuroticism | *TCTA* | 6988 | 3 | 49449639 | 49453909 | 6.61 | 4.83 | 1.97E-11 | 6.78E-07 |
| CD | Neuroticism | *THADA* | 63892 | 2 | 43457975 | 43823185 | 5.98 | 5.73 | 1.11E-09 | 4.92E-09 |
| CD | Neuroticism | *TNXB* | 7148 | 6 | 32008932 | 32077151 | 6.05 | 6.39 | 7.05E-10 | 8.08E-11 |
| CD | Neuroticism | *TRAIP* | 10293 | 3 | 49866028 | 49893992 | 5.99 | 4.76 | 1.04E-09 | 9.62E-07 |
| GERD | Neuroticism | *ABT1* | 29777 | 6 | 26597171 | 26600278 | 5.31 | 4.70 | 5.41E-08 | 1.32E-06 |
| GERD | Neuroticism | *ATP6V1G2* | 534 | 6 | 31512228 | 31514625 | 6.17 | 5.18 | 3.46E-10 | 1.12E-07 |
| GERD | Neuroticism | *BAG6* | 7917 | 6 | 31606805 | 31620953 | 5.68 | 5.96 | 6.79E-09 | 1.30E-09 |
| GERD | Neuroticism | *BTN2A1* | 11120 | 6 | 26458132 | 26476849 | 7.35 | 6.02 | 9.56E-14 | 8.59E-10 |
| GERD | Neuroticism | *BTN3A2* | 11118 | 6 | 26365387 | 26378548 | 6.18 | 5.65 | 3.29E-10 | 8.01E-09 |
| GERD | Neuroticism | *DDR1* | 780 | 6 | 30850694 | 30867933 | 5.70 | 5.47 | 5.89E-09 | 2.26E-08 |
| GERD | Neuroticism | *DRD2* | 1813 | 11 | 113280317 | 113346413 | 5.39 | 6.49 | 3.62E-08 | 4.36E-11 |
| GERD | Neuroticism | *ELAVL2* | 1993 | 9 | 23690097 | 23826342 | 4.76 | 5.08 | 9.79E-07 | 1.92E-07 |
| GERD | Neuroticism | *GPSM3* | 63940 | 6 | 32158543 | 32163300 | 4.67 | 4.57 | 1.53E-06 | 2.38E-06 |
| GERD | Neuroticism | *GRM8* | 2918 | 7 | 126078652 | 126892428 | 4.86 | 5.83 | 5.89E-07 | 2.69E-09 |
| GERD | Neuroticism | *GTF2H4* | 2968 | 6 | 30875957 | 30881883 | 5.08 | 5.05 | 1.87E-07 | 2.20E-07 |
| GERD | Neuroticism | *H1-5* | 3009 | 6 | 27834570 | 27835359 | 5.84 | 4.94 | 2.57E-09 | 3.85E-07 |
| GERD | Neuroticism | *H2AC16* | 8332 | 6 | 27833107 | 27833576 | 6.26 | 5.45 | 1.93E-10 | 2.47E-08 |
| GERD | Neuroticism | *H2BC13* | 8340 | 6 | 27775257 | 27775709 | 6.33 | 4.92 | 1.24E-10 | 4.29E-07 |
| GERD | Neuroticism | *H2BC15* | 8341 | 6 | 27805544 | 27821533 | 6.71 | 6.81 | 1.00E-11 | 5.05E-12 |
| GERD | Neuroticism | *H2BC17* | 8348 | 6 | 27861203 | 27926100 | 5.19 | 4.98 | 1.04E-07 | 3.14E-07 |
| GERD | Neuroticism | *H2BC4* | 8347 | 6 | 26113389 | 26124266 | 4.90 | 5.49 | 4.78E-07 | 2.04E-08 |
| GERD | Neuroticism | *H3C11* | 8354 | 6 | 27839623 | 27840099 | 4.71 | 5.11 | 1.21E-06 | 1.63E-07 |
| GERD | Neuroticism | *H3C12* | 8356 | 6 | 27858093 | 27858570 | 6.45 | 6.76 | 5.62E-11 | 6.80E-12 |
| GERD | Neuroticism | *H3C2* | 8358 | 6 | 26031817 | 26032288 | 5.82 | 5.19 | 2.87E-09 | 1.06E-07 |
| GERD | Neuroticism | *H3C3* | 8352 | 6 | 26045639 | 26046097 | 6.01 | 6.09 | 9.47E-10 | 5.58E-10 |
| GERD | Neuroticism | *H4C13* | 8368 | 6 | 27840926 | 27841289 | 6.74 | 6.51 | 7.72E-12 | 3.68E-11 |
| GERD | Neuroticism | *HLA-B* | 3106 | 6 | 31321649 | 31324989 | 5.65 | 5.38 | 8.08E-09 | 3.78E-08 |
| GERD | Neuroticism | *HLA-C* | 3107 | 6 | 31236526 | 31239913 | 5.82 | 4.95 | 3.00E-09 | 3.64E-07 |
| GERD | Neuroticism | *HLA-DRA* | 3122 | 6 | 32407619 | 32412823 | 5.07 | 5.32 | 2.04E-07 | 5.14E-08 |
| GERD | Neuroticism | *LAMB2* | 3913 | 3 | 49158547 | 49170599 | 5.31 | 5.07 | 5.57E-08 | 2.02E-07 |
| GERD | Neuroticism | *LSM2* | 57819 | 6 | 31765169 | 31774761 | 5.53 | 5.77 | 1.64E-08 | 3.91E-09 |
| GERD | Neuroticism | *MSH5* | 4439 | 6 | 31707725 | 31730455 | 6.41 | 6.53 | 7.41E-11 | 3.29E-11 |
| GERD | Neuroticism | *MUCL3* | 135656 | 6 | 30908777 | 30921998 | 5.88 | 5.45 | 2.03E-09 | 2.51E-08 |
| GERD | Neuroticism | *NCAM1* | 4684 | 11 | 112831969 | 113149158 | 6.33 | 6.15 | 1.24E-10 | 3.78E-10 |
| GERD | Neuroticism | *OR2B2* | 81697 | 6 | 27878963 | 27880174 | 5.51 | 5.07 | 1.80E-08 | 1.98E-07 |
| GERD | Neuroticism | *PGBD1* | 84547 | 6 | 28249314 | 28270326 | 5.82 | 5.19 | 2.87E-09 | 1.07E-07 |
| GERD | Neuroticism | *PRRC2A* | 7916 | 6 | 31588450 | 31605554 | 5.97 | 6.04 | 1.20E-09 | 7.95E-10 |
| GERD | Neuroticism | *RABGAP1L* | 9910 | 1 | 174128552 | 174964445 | 4.70 | 5.04 | 1.30E-06 | 2.31E-07 |
| GERD | Neuroticism | *RBM5* | 10181 | 3 | 50126341 | 50156397 | 5.11 | 4.56 | 1.64E-07 | 2.62E-06 |
| GERD | Neuroticism | *SFTA2* | 389376 | 6 | 30899127 | 30899952 | 5.83 | 6.02 | 2.72E-09 | 8.75E-10 |
| GERD | Neuroticism | *SNHG32* | 50854 | 6 | 31802693 | 31807541 | 5.54 | 5.44 | 1.51E-08 | 2.60E-08 |
| GERD | Neuroticism | *TNXB* | 7148 | 6 | 32008932 | 32077151 | 4.90 | 5.48 | 4.81E-07 | 2.12E-08 |
| GERD | Neuroticism | *TRIM26* | 7726 | 6 | 30152232 | 30181271 | 5.58 | 5.47 | 1.20E-08 | 2.23E-08 |
| GERD | Neuroticism | *TRIM31* | 11074 | 6 | 30070674 | 30080867 | 5.02 | 4.91 | 2.53E-07 | 4.63E-07 |
| GERD | Neuroticism | *TRIM39* | 56658 | 6 | 30294227 | 30311506 | 4.79 | 4.70 | 8.26E-07 | 1.28E-06 |
| GERD | Neuroticism | *UGP2* | 7360 | 2 | 64068084 | 64118696 | 5.20 | 5.32 | 9.96E-08 | 5.14E-08 |
| GERD | Neuroticism | *VARS1* | 7407 | 6 | 31745295 | 31763712 | 5.34 | 5.66 | 4.54E-08 | 7.39E-09 |
| GERD | Neuroticism | *VWA7* | 80737 | 6 | 31733178 | 31745108 | 5.80 | 6.01 | 3.38E-09 | 9.24E-10 |
| GERD | Neuroticism | *ZKSCAN4* | 387032 | 6 | 28212404 | 28227030 | 5.40 | 5.55 | 3.26E-08 | 1.41E-08 |
| GERD | Neuroticism | *ZKSCAN8* | 7745 | 6 | 28109688 | 28127250 | 5.26 | 5.11 | 7.06E-08 | 1.64E-07 |
| GERD | Neuroticism | *ZNF311* | 282890 | 6 | 28962562 | 28973037 | 5.19 | 4.77 | 1.05E-07 | 9.43E-07 |
| GERD | Neuroticism | *ZSCAN16* | 80345 | 6 | 28092334 | 28097864 | 4.88 | 5.07 | 5.18E-07 | 1.98E-07 |
| GERD | Neuroticism | *ZSCAN26* | 7741 | 6 | 28234788 | 28246001 | 5.62 | 4.97 | 9.33E-09 | 3.41E-07 |
| IBD | Neuroticism | *CFB* | 629 | 6 | 31913721 | 31919861 | 7.38 | 4.63 | 8.09E-14 | 1.83E-06 |
| IBD | Neuroticism | *EHMT2* | 10919 | 6 | 31847536 | 31865464 | 5.61 | 5.05 | 1.02E-08 | 2.25E-07 |
| IBD | Neuroticism | *H2AC16* | 8332 | 6 | 27833107 | 27833576 | 4.61 | 5.08 | 2.05E-06 | 1.93E-07 |
| IBD | Neuroticism | *H2BC13* | 8340 | 6 | 27775257 | 27775709 | 4.60 | 5.11 | 2.10E-06 | 1.58E-07 |
| IBD | Neuroticism | *H2BC15* | 8341 | 6 | 27805544 | 27821533 | 4.67 | 5.41 | 1.49E-06 | 3.16E-08 |
| IBD | Neuroticism | *HLA-DQB1* | 3119 | 6 | 32627241 | 32634466 | 7.12 | 5.09 | 5.22E-13 | 1.83E-07 |
| IBD | Neuroticism | *HLA-DRA* | 3122 | 6 | 32407619 | 32412823 | 5.30 | 5.29 | 5.70E-08 | 6.02E-08 |
| IBD | Neuroticism | *MSH5* | 4439 | 6 | 31707725 | 31730455 | 4.68 | 5.37 | 1.47E-06 | 3.93E-08 |
| IBD | Neuroticism | *PUS10* | 150962 | 2 | 61167548 | 61245365 | 5.87 | 4.90 | 2.21E-09 | 4.73E-07 |
| IBD | Neuroticism | *TNXB* | 7148 | 6 | 32008932 | 32077151 | 6.09 | 5.13 | 5.79E-10 | 1.44E-07 |
| IBD | Neuroticism | *TRIM31* | 11074 | 6 | 30070674 | 30080867 | 6.26 | 5.27 | 1.89E-10 | 6.84E-08 |
| IBD | Neuroticism | *VWA7* | 80737 | 6 | 31733178 | 31745108 | 5.30 | 5.37 | 5.79E-08 | 4.03E-08 |
| IBS | Neuroticism | *ACO2* | 50 | 22 | 41865099 | 41924993 | 5.02 | 4.91 | 2.65E-07 | 4.61E-07 |
| IBS | Neuroticism | *AIF1* | 199 | 6 | 31582969 | 31584798 | 4.63 | 4.71 | 1.80E-06 | 1.21E-06 |
| IBS | Neuroticism | *ANKK1* | 255239 | 11 | 113258513 | 113271140 | 5.25 | 4.72 | 7.76E-08 | 1.17E-06 |
| IBS | Neuroticism | *ATF6B* | 1388 | 6 | 32083045 | 32096017 | 5.72 | 5.74 | 5.49E-09 | 4.80E-09 |
| IBS | Neuroticism | *BAG6* | 7917 | 6 | 31606805 | 31620953 | 5.67 | 5.58 | 7.25E-09 | 1.20E-08 |
| IBS | Neuroticism | *CADM2* | 253559 | 3 | 85008133 | 86123579 | 6.20 | 5.71 | 2.89E-10 | 5.77E-09 |
| IBS | Neuroticism | *CELF4* | 56853 | 18 | 34823003 | 35146000 | 6.48 | 5.25 | 4.55E-11 | 7.72E-08 |
| IBS | Neuroticism | *DLST* | 1743 | 14 | 75348594 | 75370450 | 6.22 | 5.75 | 2.55E-10 | 4.42E-09 |
| IBS | Neuroticism | *DRD2* | 1813 | 11 | 113280317 | 113346413 | 7.05 | 6.26 | 8.94E-13 | 1.92E-10 |
| IBS | Neuroticism | *EP300* | 2033 | 22 | 41488614 | 41576081 | 5.02 | 4.58 | 2.55E-07 | 2.38E-06 |
| IBS | Neuroticism | *FAM120A* | 23196 | 9 | 96214173 | 96328397 | 6.31 | 5.96 | 1.38E-10 | 1.22E-09 |
| IBS | Neuroticism | *FKBPL* | 63943 | 6 | 32096484 | 32098067 | 4.92 | 4.64 | 4.41E-07 | 1.75E-06 |
| IBS | Neuroticism | *GRM8* | 2918 | 7 | 126078652 | 126892428 | 5.84 | 4.93 | 2.63E-09 | 4.18E-07 |
| IBS | Neuroticism | *HLA-C* | 3107 | 6 | 31236526 | 31239913 | 4.90 | 4.56 | 4.77E-07 | 2.58E-06 |
| IBS | Neuroticism | *IL20RB* | 53833 | 3 | 136676707 | 136729927 | 4.75 | 4.56 | 1.03E-06 | 2.58E-06 |
| IBS | Neuroticism | *NCAM1* | 4684 | 11 | 112831969 | 113149158 | 6.72 | 6.36 | 9.30E-12 | 9.90E-11 |
| IBS | Neuroticism | *PCCB* | 5096 | 3 | 135969167 | 136056737 | 5.54 | 5.53 | 1.51E-08 | 1.56E-08 |
| IBS | Neuroticism | *PHF5A* | 84844 | 22 | 41855721 | 41864708 | 4.96 | 4.91 | 3.49E-07 | 4.61E-07 |
| IBS | Neuroticism | *POLR3H* | 171568 | 22 | 41921803 | 41940479 | 5.02 | 4.91 | 2.55E-07 | 4.58E-07 |
| IBS | Neuroticism | *PROX2* | 283571 | 14 | 75319736 | 75330562 | 5.59 | 5.10 | 1.12E-08 | 1.73E-07 |
| IBS | Neuroticism | *PRRC2A* | 7916 | 6 | 31588450 | 31605554 | 4.97 | 4.68 | 3.34E-07 | 1.43E-06 |
| IBS | Neuroticism | *RABGAP1L* | 9910 | 1 | 174128552 | 174964445 | 4.78 | 4.61 | 8.59E-07 | 2.04E-06 |
| IBS | Neuroticism | *RANGAP1* | 5905 | 22 | 41640781 | 41682255 | 6.59 | 6.19 | 2.26E-11 | 3.02E-10 |
| IBS | Neuroticism | *RERE* | 473 | 1 | 8412464 | 8877699 | 4.83 | 4.62 | 6.91E-07 | 1.97E-06 |
| IBS | Neuroticism | *SKIV2L* | 6499 | 6 | 31926581 | 31937532 | 5.17 | 4.70 | 1.16E-07 | 1.30E-06 |
| IBS | Neuroticism | *STAG1* | 10274 | 3 | 136055077 | 136471245 | 5.87 | 5.66 | 2.12E-09 | 7.37E-09 |
| IBS | Neuroticism | *TEF* | 7008 | 22 | 41763337 | 41795332 | 5.22 | 5.00 | 9.00E-08 | 2.80E-07 |
| IBS | Neuroticism | *TMEM106B* | 54664 | 7 | 12250848 | 12276890 | 5.29 | 5.03 | 6.27E-08 | 2.42E-07 |
| IBS | Neuroticism | *TMEM161B* | 153396 | 5 | 87485450 | 87564665 | 5.20 | 4.88 | 1.01E-07 | 5.32E-07 |
| IBS | Neuroticism | *TNXB* | 7148 | 6 | 32008932 | 32077151 | 6.15 | 5.32 | 3.77E-10 | 5.15E-08 |
| IBS | Neuroticism | *TOB2* | 10766 | 22 | 41829492 | 41844234 | 5.07 | 4.91 | 1.97E-07 | 4.47E-07 |
| IBS | Neuroticism | *TTC12* | 54970 | 11 | 113185251 | 113244345 | 5.90 | 5.33 | 1.84E-09 | 4.91E-08 |
| IBS | Neuroticism | *YLPM1* | 56252 | 14 | 75230025 | 75304013 | 5.77 | 5.34 | 4.07E-09 | 4.59E-08 |
| IBS | Neuroticism | *ZC3H7B* | 23264 | 22 | 41697507 | 41756151 | 5.91 | 5.75 | 1.67E-09 | 4.48E-09 |
| PUD | Neuroticism | *FBXL17* | 64839 | 5 | 107194734 | 107718080 | 4.89 | 5.25 | 5.14E-07 | 7.47E-08 |
| PUD | Neuroticism | *H3C12* | 8356 | 6 | 27858093 | 27858570 | 4.64 | 5.05 | 1.76E-06 | 2.17E-07 |
| PUD | Neuroticism | *LY6K* | 54742 | 8 | 143781529 | 143785588 | 5.97 | 4.98 | 1.21E-09 | 3.22E-07 |
| PUD | Neuroticism | *NCAM1* | 4684 | 11 | 112831969 | 113149158 | 4.63 | 5.04 | 1.85E-06 | 2.36E-07 |
| PUD | Neuroticism | *PAX6* | 5080 | 11 | 31806340 | 31839509 | 4.97 | 5.44 | 3.37E-07 | 2.70E-08 |
| PUD | Neuroticism | *PSCA* | 8000 | 8 | 143751726 | 143764145 | 7.02 | 5.05 | 1.09E-12 | 2.17E-07 |
| PUD | Neuroticism | *THEM6* | 51337 | 8 | 143808621 | 143818350 | 6.27 | 4.67 | 1.84E-10 | 1.49E-06 |
| UC | Neuroticism | *AMT* | 275 | 3 | 49454211 | 49460111 | 5.32 | 4.67 | 5.19E-08 | 1.52E-06 |
| UC | Neuroticism | *APEH* | 327 | 3 | 49711427 | 49720936 | 6.85 | 4.84 | 3.59E-12 | 6.44E-07 |
| UC | Neuroticism | *BSN* | 8927 | 3 | 49591922 | 49708982 | 6.32 | 5.32 | 1.33E-10 | 5.05E-08 |
| UC | Neuroticism | *CFB* | 629 | 6 | 31913721 | 31919861 | 7.77 | 5.34 | 3.83E-15 | 4.56E-08 |
| UC | Neuroticism | *CREM* | 1390 | 10 | 35415769 | 35501886 | 5.09 | 4.61 | 1.81E-07 | 2.02E-06 |
| UC | Neuroticism | *CUL2* | 8453 | 10 | 35297479 | 35379570 | 5.15 | 4.69 | 1.29E-07 | 1.36E-06 |
| UC | Neuroticism | *DAG1* | 1605 | 3 | 49506136 | 49573051 | 5.36 | 5.09 | 4.15E-08 | 1.81E-07 |
| UC | Neuroticism | *GPX1* | 2876 | 3 | 49394609 | 49395791 | 5.99 | 5.85 | 1.05E-09 | 2.44E-09 |
| UC | Neuroticism | *HLA-DQA1* | 3117 | 6 | 32605169 | 32612152 | 7.49 | 7.13 | 3.42E-14 | 5.11E-13 |
| UC | Neuroticism | *HLA-DQB1* | 3119 | 6 | 32627241 | 32634466 | 7.88 | 6.74 | 1.61E-15 | 8.09E-12 |
| UC | Neuroticism | *HLA-DRB1* | 3123 | 6 | 32546546 | 32557613 | 6.89 | 7.87 | 2.72E-12 | 1.78E-15 |
| UC | Neuroticism | *MST1* | 4485 | 3 | 49721380 | 49726196 | 6.50 | 5.64 | 4.00E-11 | 8.58E-09 |
| UC | Neuroticism | *NICN1* | 84276 | 3 | 49459766 | 49466777 | 5.24 | 5.44 | 8.11E-08 | 2.63E-08 |
| UC | Neuroticism | *PSORS1C1* | 170679 | 6 | 31082608 | 31107869 | 5.51 | 5.21 | 1.82E-08 | 9.68E-08 |
| UC | Neuroticism | *PUS10* | 150962 | 2 | 61167548 | 61245365 | 7.06 | 5.77 | 8.36E-13 | 4.00E-09 |
| UC | Neuroticism | *RHOA* | 387 | 3 | 49396569 | 49449526 | 5.28 | 5.23 | 6.46E-08 | 8.34E-08 |
| UC | Neuroticism | *RNF123* | 63891 | 3 | 49726950 | 49758962 | 4.94 | 5.17 | 3.99E-07 | 1.16E-07 |
| UC | Neuroticism | *STK19* | 8859 | 6 | 31938952 | 31949223 | 5.21 | 7.31 | 9.65E-08 | 1.38E-13 |
| UC | Neuroticism | *TCTA* | 6988 | 3 | 49449639 | 49453909 | 5.91 | 5.86 | 1.66E-09 | 2.39E-09 |
| UC | Neuroticism | *TNXB* | 7148 | 6 | 32008932 | 32077151 | 5.81 | 6.54 | 3.07E-09 | 3.05E-11 |
| GERD | Worry | *ATP6V1G2* | 534 | 6 | 31512228 | 31514625 | 6.49 | 5.82 | 4.42E-11 | 3.01E-09 |
| GERD | Worry | *BAG6* | 7917 | 6 | 31606805 | 31620953 | 5.86 | 6.61 | 2.33E-09 | 1.95E-11 |
| GERD | Worry | *BORCS7* | 119032 | 10 | 104613967 | 104624718 | 4.64 | 5.60 | 1.74E-06 | 1.09E-08 |
| GERD | Worry | *BTN2A1* | 11120 | 6 | 26458132 | 26476849 | 7.43 | 6.37 | 5.58E-14 | 9.63E-11 |
| GERD | Worry | *BTN3A2* | 11118 | 6 | 26365387 | 26378548 | 6.30 | 6.01 | 1.49E-10 | 9.15E-10 |
| GERD | Worry | *C6orf136* | 221545 | 6 | 30614816 | 30620987 | 5.44 | 4.74 | 2.70E-08 | 1.08E-06 |
| GERD | Worry | *CSNK2B* | 1460 | 6 | 31632995 | 31637844 | 4.62 | 5.55 | 1.88E-06 | 1.43E-08 |
| GERD | Worry | *DDR1* | 780 | 6 | 30850694 | 30867933 | 5.62 | 5.40 | 9.47E-09 | 3.34E-08 |
| GERD | Worry | *GPSM3* | 63940 | 6 | 32158543 | 32163300 | 4.68 | 4.84 | 1.43E-06 | 6.41E-07 |
| GERD | Worry | *GTF2H4* | 2968 | 6 | 30875957 | 30881883 | 4.99 | 4.98 | 3.04E-07 | 3.25E-07 |
| GERD | Worry | *H1-5* | 3009 | 6 | 27834570 | 27835359 | 5.69 | 5.07 | 6.36E-09 | 2.00E-07 |
| GERD | Worry | *H2AC16* | 8332 | 6 | 27833107 | 27833576 | 6.13 | 6.11 | 4.37E-10 | 5.00E-10 |
| GERD | Worry | *H2BC13* | 8340 | 6 | 27775257 | 27775709 | 6.23 | 4.94 | 2.37E-10 | 3.99E-07 |
| GERD | Worry | *H2BC15* | 8341 | 6 | 27805544 | 27821533 | 6.58 | 6.65 | 2.32E-11 | 1.44E-11 |
| GERD | Worry | *H2BC17* | 8348 | 6 | 27861203 | 27926100 | 5.10 | 4.82 | 1.74E-07 | 7.10E-07 |
| GERD | Worry | *H2BC4* | 8347 | 6 | 26113389 | 26124266 | 4.68 | 5.57 | 1.46E-06 | 1.27E-08 |
| GERD | Worry | *H3C12* | 8356 | 6 | 27858093 | 27858570 | 6.32 | 6.61 | 1.31E-10 | 1.86E-11 |
| GERD | Worry | *H3C2* | 8358 | 6 | 26031817 | 26032288 | 5.69 | 4.96 | 6.21E-09 | 3.52E-07 |
| GERD | Worry | *H3C3* | 8352 | 6 | 26045639 | 26046097 | 5.89 | 6.06 | 1.96E-09 | 6.88E-10 |
| GERD | Worry | *H4C13* | 8368 | 6 | 27840926 | 27841289 | 6.75 | 6.56 | 7.35E-12 | 2.63E-11 |
| GERD | Worry | *H4C3* | 8364 | 6 | 26104176 | 26104565 | 4.81 | 4.60 | 7.55E-07 | 2.11E-06 |
| GERD | Worry | *HLA-B* | 3106 | 6 | 31321649 | 31324989 | 5.63 | 4.82 | 8.98E-09 | 7.08E-07 |
| GERD | Worry | *HLA-C* | 3107 | 6 | 31236526 | 31239913 | 5.87 | 5.44 | 2.12E-09 | 2.74E-08 |
| GERD | Worry | *HLA-DRA* | 3122 | 6 | 32407619 | 32412823 | 5.02 | 5.53 | 2.52E-07 | 1.63E-08 |
| GERD | Worry | *LSM2* | 57819 | 6 | 31765169 | 31774761 | 5.82 | 6.66 | 3.01E-09 | 1.41E-11 |
| GERD | Worry | *MSH5* | 4439 | 6 | 31707725 | 31730455 | 6.56 | 7.23 | 2.60E-11 | 2.40E-13 |
| GERD | Worry | *MUCL3* | 135656 | 6 | 30908777 | 30921998 | 5.81 | 5.31 | 3.06E-09 | 5.47E-08 |
| GERD | Worry | *NCAM1* | 4684 | 11 | 112831969 | 113149158 | 5.85 | 6.07 | 2.48E-09 | 6.24E-10 |
| GERD | Worry | *OR2B2* | 81697 | 6 | 27878963 | 27880174 | 6.11 | 6.33 | 5.00E-10 | 1.25E-10 |
| GERD | Worry | *PGBD1* | 84547 | 6 | 28249314 | 28270326 | 5.75 | 4.91 | 4.41E-09 | 4.45E-07 |
| GERD | Worry | *PRRC2A* | 7916 | 6 | 31588450 | 31605554 | 6.28 | 7.09 | 1.69E-10 | 6.74E-13 |
| GERD | Worry | *SFTA2* | 389376 | 6 | 30899127 | 30899952 | 6.67 | 5.97 | 1.30E-11 | 1.22E-09 |
| GERD | Worry | *SNHG32* | 50854 | 6 | 31802693 | 31807541 | 5.80 | 6.08 | 3.30E-09 | 6.08E-10 |
| GERD | Worry | *TNXB* | 7148 | 6 | 32008932 | 32077151 | 4.80 | 5.69 | 8.06E-07 | 6.44E-09 |
| GERD | Worry | *TRIM26* | 7726 | 6 | 30152232 | 30181271 | 5.52 | 5.39 | 1.70E-08 | 3.54E-08 |
| GERD | Worry | *TRIM31* | 11074 | 6 | 30070674 | 30080867 | 5.03 | 5.12 | 2.44E-07 | 1.52E-07 |
| GERD | Worry | *TUBB* | 203068 | 6 | 30688012 | 30693199 | 4.76 | 5.17 | 9.87E-07 | 1.18E-07 |
| GERD | Worry | *UGP2* | 7360 | 2 | 64068084 | 64118696 | 4.88 | 4.64 | 5.28E-07 | 1.78E-06 |
| GERD | Worry | *VARS1* | 7407 | 6 | 31745295 | 31763712 | 5.53 | 6.58 | 1.58E-08 | 2.30E-11 |
| GERD | Worry | *VWA7* | 80737 | 6 | 31733178 | 31745108 | 6.03 | 6.66 | 8.22E-10 | 1.41E-11 |
| GERD | Worry | *ZKSCAN4* | 387032 | 6 | 28212404 | 28227030 | 5.28 | 5.39 | 6.64E-08 | 3.45E-08 |
| GERD | Worry | *ZKSCAN8* | 7745 | 6 | 28109688 | 28127250 | 5.21 | 5.08 | 9.66E-08 | 1.94E-07 |
| GERD | Worry | *ZSCAN16* | 80345 | 6 | 28092334 | 28097864 | 4.80 | 5.08 | 7.75E-07 | 1.93E-07 |
| GERD | Worry | *ZSCAN26* | 7741 | 6 | 28234788 | 28246001 | 5.54 | 4.78 | 1.52E-08 | 8.66E-07 |
| IBS | Worry | *AIF1* | 199 | 6 | 31582969 | 31584798 | 5.33 | 5.12 | 5.03E-08 | 1.50E-07 |
| IBS | Worry | *ALAS1* | 211 | 3 | 52232099 | 52248343 | 5.39 | 5.39 | 3.47E-08 | 3.48E-08 |
| IBS | Worry | *AS3MT* | 57412 | 10 | 104629183 | 104661656 | 5.85 | 4.62 | 2.52E-09 | 1.90E-06 |
| IBS | Worry | *ATF6B* | 1388 | 6 | 32083045 | 32096017 | 6.25 | 5.62 | 2.00E-10 | 9.82E-09 |
| IBS | Worry | *BAG6* | 7917 | 6 | 31606805 | 31620953 | 6.71 | 5.93 | 9.79E-12 | 1.51E-09 |
| IBS | Worry | *BORCS7* | 119032 | 10 | 104613967 | 104624718 | 5.51 | 4.87 | 1.84E-08 | 5.51E-07 |
| IBS | Worry | *CADM2* | 253559 | 3 | 85008133 | 86123579 | 9.40 | 8.79 | 2.86E-21 | 7.53E-19 |
| IBS | Worry | *CREB3L4* | 148327 | 1 | 153940315 | 153946840 | 5.23 | 4.85 | 8.46E-08 | 6.08E-07 |
| IBS | Worry | *CSNK2B* | 1460 | 6 | 31632995 | 31637844 | 5.32 | 4.73 | 5.32E-08 | 1.14E-06 |
| IBS | Worry | *CYP17A1* | 1586 | 10 | 104590288 | 104597290 | 5.08 | 4.60 | 1.86E-07 | 2.08E-06 |
| IBS | Worry | *DLST* | 1743 | 14 | 75348594 | 75370450 | 6.33 | 5.96 | 1.23E-10 | 1.24E-09 |
| IBS | Worry | *DRD2* | 1813 | 11 | 113280317 | 113346413 | 5.51 | 5.18 | 1.75E-08 | 1.09E-07 |
| IBS | Worry | *FAM120A* | 23196 | 9 | 96214173 | 96328397 | 5.89 | 5.20 | 1.97E-09 | 1.02E-07 |
| IBS | Worry | *FKBPL* | 63943 | 6 | 32096484 | 32098067 | 5.52 | 4.88 | 1.68E-08 | 5.24E-07 |
| IBS | Worry | *GATAD2B* | 57459 | 1 | 153777201 | 153895451 | 5.11 | 4.86 | 1.57E-07 | 5.87E-07 |
| IBS | Worry | *HLA-C* | 3107 | 6 | 31236526 | 31239913 | 5.16 | 4.91 | 1.25E-07 | 4.56E-07 |
| IBS | Worry | *IL20RB* | 53833 | 3 | 136676707 | 136729927 | 4.92 | 4.89 | 4.29E-07 | 5.05E-07 |
| IBS | Worry | *MSH5* | 4439 | 6 | 31707725 | 31730455 | 5.58 | 4.66 | 1.20E-08 | 1.60E-06 |
| IBS | Worry | *NCAM1* | 4684 | 11 | 112831969 | 113149158 | 6.53 | 6.02 | 3.34E-11 | 8.54E-10 |
| IBS | Worry | *PCCB* | 5096 | 3 | 135969167 | 136056737 | 5.61 | 5.42 | 1.00E-08 | 3.01E-08 |
| IBS | Worry | *PROX2* | 283571 | 14 | 75319736 | 75330562 | 5.51 | 5.34 | 1.79E-08 | 4.70E-08 |
| IBS | Worry | *PRRC2A* | 7916 | 6 | 31588450 | 31605554 | 6.06 | 5.13 | 6.73E-10 | 1.41E-07 |
| IBS | Worry | *RANGAP1* | 5905 | 22 | 41640781 | 41682255 | 6.22 | 5.53 | 2.49E-10 | 1.58E-08 |
| IBS | Worry | *SKIV2L* | 6499 | 6 | 31926581 | 31937532 | 5.60 | 4.96 | 1.07E-08 | 3.46E-07 |
| IBS | Worry | *STAG1* | 10274 | 3 | 136055077 | 136471245 | 5.70 | 5.39 | 6.12E-09 | 3.48E-08 |
| IBS | Worry | *STK19* | 8859 | 6 | 31938952 | 31949223 | 5.04 | 4.79 | 2.28E-07 | 8.27E-07 |
| IBS | Worry | *TLR9* | 54106 | 3 | 52255096 | 52260179 | 5.63 | 4.56 | 9.09E-09 | 2.58E-06 |
| IBS | Worry | *TMEM161B* | 153396 | 5 | 87485450 | 87564665 | 5.03 | 4.79 | 2.44E-07 | 8.44E-07 |
| IBS | Worry | *TNXB* | 7148 | 6 | 32008932 | 32077151 | 6.55 | 5.31 | 2.79E-11 | 5.44E-08 |
| IBS | Worry | *VWA7* | 80737 | 6 | 31733178 | 31745108 | 6.27 | 5.07 | 1.84E-10 | 1.97E-07 |
| IBS | Worry | *YLPM1* | 56252 | 14 | 75230025 | 75304013 | 5.71 | 5.61 | 5.69E-09 | 1.02E-08 |
| IBS | Worry | *ZC3H7B* | 23264 | 22 | 41697507 | 41756151 | 5.40 | 5.06 | 3.38E-08 | 2.05E-07 |
| PUD | Worry | *FIGN* | 55137 | 2 | 164461447 | 164592618 | 4.64 | 4.92 | 1.73E-06 | 4.36E-07 |
| PUD | Worry | *LY6K* | 54742 | 8 | 143781529 | 143785588 | 6.37 | 4.85 | 9.58E-11 | 6.32E-07 |
| PUD | Worry | *THEM6* | 51337 | 8 | 143808621 | 143818350 | 6.39 | 4.55 | 8.06E-11 | 2.72E-06 |
| UC | Worry | *CADM2* | 253559 | 3 | 85008133 | 86123579 | 5.03 | 6.21 | 2.39E-07 | 2.65E-10 |
| UC | Worry | *CFB* | 629 | 6 | 31913721 | 31919861 | 7.72 | 5.14 | 6.05E-15 | 1.41E-07 |
| UC | Worry | *EHMT2* | 10919 | 6 | 31847536 | 31865464 | 7.04 | 4.78 | 9.91E-13 | 8.95E-07 |
| UC | Worry | *GPANK1* | 7918 | 6 | 31629006 | 31634060 | 6.11 | 5.64 | 5.00E-10 | 8.47E-09 |
| UC | Worry | *HLA-DOB* | 3112 | 6 | 32780540 | 32784825 | 5.60 | 4.72 | 1.04E-08 | 1.17E-06 |
| UC | Worry | *HLA-DQA1* | 3117 | 6 | 32605169 | 32612152 | 6.11 | 6.57 | 5.00E-10 | 2.57E-11 |
| UC | Worry | *HLA-DQB1* | 3119 | 6 | 32627241 | 32634466 | 6.11 | 6.46 | 5.00E-10 | 5.18E-11 |
| UC | Worry | *HLA-DRB1* | 3123 | 6 | 32546546 | 32557613 | 7.80 | 6.79 | 3.05E-15 | 5.54E-12 |
| UC | Worry | *HSPA1L* | 3305 | 6 | 31777396 | 31790093 | 5.95 | 5.14 | 1.33E-09 | 1.40E-07 |
| UC | Worry | *OR5B17* | 219965 | 11 | 58125598 | 58126542 | 4.68 | 4.79 | 1.43E-06 | 8.18E-07 |
| UC | Worry | *OTUD3* | 23252 | 1 | 20208356 | 20239438 | 7.76 | 5.17 | 4.27E-15 | 1.20E-07 |
| UC | Worry | *PSORS1C1* | 170679 | 6 | 31082608 | 31107869 | 5.24 | 4.61 | 8.20E-08 | 2.01E-06 |
| UC | Worry | *PUS10* | 150962 | 2 | 61167548 | 61245365 | 6.11 | 5.86 | 5.00E-10 | 2.29E-09 |
| UC | Worry | *SANBR* | 84542 | 2 | 61293005 | 61365169 | 4.72 | 4.61 | 1.20E-06 | 2.02E-06 |
| UC | Worry | *SFMBT1* | 51460 | 3 | 52933221 | 53080089 | 5.31 | 5.87 | 5.50E-08 | 2.21E-09 |
| UC | Worry | *TNXB* | 7148 | 6 | 32008932 | 32077151 | 5.40 | 6.78 | 3.39E-08 | 6.03E-12 |

**Table S10**: Top 10 Tissue-specific gene expression using JTI model.

| **Gene** | **Trait 1** | **Trait 2** | **Tissue code** |
| --- | --- | --- | --- |
| *C4A* | CD | Neuroticism | T1,T21,T23 |
| *C4A* | GERD | Neuroticism | T1,T2,T3,T4,T5,T6,T7,T8,T9,T10,T11,T12,T13,T14,T15,T16,T17,T18,T19,T20,T21,T22,T23,T24,T25 |
| *C4A* | GERD | Worry | T1,T2,T3,T4,T5,T6,T7,T8,T9,T10,T11,T12,T13,T14,T15,T16,T17,T18,T19,T20,T21,T22,T23,T24,T25 |
| *C4A* | IBD | Neuroticism | T1,T2,T3,T4,T5,T6,T7,T8,T9,T10,T11,T12,T13,T14,T15,T16,T17,T18,T19,T20,T21,T22,T23,T24,T25 |
| *C4A* | UC | Neuroticism | T2,T3,T4,T5,T6,T7,T8,T9,T10,T11,T12,T13,T15,T16,T17,T18,T20,T22,T24,T25 |
| *C4A* | UC | Worry | T2,T3,T4,T5,T6,T7,T8,T9,T10,T11,T12,T13,T15,T25 |
| *BTN3A2* | GERD | Neuroticism | T1,T2,T4,T5,T6,T8,T9,T13,T15,T16,T17,T18,T19,T20,T21,T22,T23,T24,T25 |
| *BTN3A2* | GERD | Worry | T1,T2,T3,T4,T5,T6,T8,T9,T10,T13,T14,T15,T16,T17,T18,T19,T20,T21,T22,T23,T24,T25 |
| *BTN3A2* | IBS | Worry | T15,T21 |
| *MSH5* | GERD | Neuroticism | T2,T7,T8,T10,T11,T12,T13,T16,T17,T18,T21,T22,T24 |
| *MSH5* | GERD | Worry | T2,T7,T8,T10,T11,T12,T13,T16,T17,T18,T21,T22,T24 |
| *MSH5* | IBD | Neuroticism | T10 |
| *MSH5* | IBS | Neuroticism | T2,T8,T10,T11,T12,T13,T16,T17,T18,T20,T21,T22,T24 |
| *MSH5* | IBS | Worry | T2,T8,T10,T11,T12,T13,T16,T17,T18,T20,T21,T22,T24 |
| *MSH5* | PUD | Neuroticism | T17 |
| *FLOT1* | GERD | Neuroticism | T1,T2,T3,T4,T5,T6,T7,T8,T9,T10,T11,T12,T17,T18,T19,T20,T21,T22,T23,T24 |
| *FLOT1* | GERD | Worry | T1,T2,T3,T4,T5,T6,T7,T8,T9,T10,T11,T12,T17,T18,T19,T20,T21,T22,T23,T24 |
| *FLOT1* | IBS | Neuroticism | T1,T5,T6 |
| *FLOT1* | IBS | Worry | T1,T3,T5,T6,T7,T8,T9,T10,T12,T18,T21,T22 |
| *PCCB* | IBS | Neuroticism | T1,T2,T3,T4,T5,T6,T7,T8,T9,T10,T11,T12,T13,T14,T15,T16,T19,T20,T22,T23,T24,T25 |
| *PCCB* | IBS | Worry | T1,T2,T3,T4,T5,T6,T7,T8,T9,T10,T11,T12,T14,T15,T16,T19,T20,T22,T23,T24,T25 |
| *ZSCAN26* | GERD | Neuroticism | T1,T2,T4,T6,T7,T8,T11,T12,T13,T14,T15,T16,T17,T18,T19,T20,T21,T22,T24,T25 |
| *ZSCAN26* | GERD | Worry | T1,T2,T4,T6,T7,T8,T12,T14,T15,T16,T17,T18,T19,T21,T22,T24,T25 |
| *ZSCAN9* | GERD | Neuroticism | T2,T3,T4,T5,T8,T9,T12,T13,T16,T18,T22 |
| *ZSCAN9* | GERD | Worry | T2,T3,T4,T5,T8,T9,T12,T13,T16,T18,T22 |
| *ZSCAN9* | IBS | Neuroticism | T2,T3,T4,T9,T12,T13,T22 |
| *ZSCAN9* | IBS | Worry | T2,T3,T4,T5,T8,T9,T12 |
| *LNCOC1* | PUD | Neuroticism | T1,T2,T3,T4,T5,T6,T7,T8,T9,T10,T11,T12,T13,T14,T15,T16,T17,T18,T19,T20,T21,T22,T23,T24 |
| *LNCOC1* | PUD | Worry | T1,T2,T3,T4,T5,T6,T7,T8,T9,T10,T11,T12,T13,T14,T15,T16,T17,T18,T19,T20,T21,T22,T23,T24 |
| *RERE-AS1* | IBS | Neuroticism | T2,T4,T5,T6,T7,T10,T11,T12,T15,T16,T17,T18,T19,T20,T21,T23,T24,T25 |
| *RERE-AS1* | IBS | Worry | T2,T4,T6,T7,T10,T11,T12,T15,T21 |
| *PRSS16* | GERD | Neuroticism | T5,T6,T7,T8,T10,T11,T12,T16,T17,T18,T19,T22,T24 |
| *PRSS16* | GERD | Worry | T5,T6,T7,T8,T10,T11,T12,T16,T17,T18,T19,T20,T22,T24 |

The tissue code was shown in Table S2.

**Table S11**: Instrumental variables for depression or dysthymia.

| **rsid** | **A1** | **A2** | **beta** | **eaf** | **N** | **SE** | ***p*** | **Confounding phenotype** |
| --- | --- | --- | --- | --- | --- | --- | --- | --- |
| **Instrumental variables** | | | | | | | | |
| rs1433574 | C | A | 0.043 | 0.274 | 297542 | 0.008 | 2.18E-08 | - |
| rs13006508 | G | A | -0.039 | 0.521 | 297542 | 0.007 | 1.27E-08 | - |
| rs6795061 | C | T | 0.049 | 0.656 | 297542 | 0.007 | 1.53E-11 | - |
| rs9917761 | C | T | 0.049 | 0.182 | 297542 | 0.009 | 4.31E-08 | - |
| rs9809577 | A | C | -0.067 | 0.824 | 297542 | 0.009 | 8.14E-14 | - |
| rs6798590 | A | T | -0.040 | 0.451 | 297542 | 0.007 | 1.25E-08 | - |
| rs10154992 | T | C | -0.054 | 0.785 | 297542 | 0.008 | 9.86E-11 | - |
| rs147831713 | C | A | 0.052 | 0.348 | 297542 | 0.007 | 6.21E-13 | - |
| rs6891234 | A | C | 0.044 | 0.357 | 297542 | 0.007 | 9.22E-10 | - |
| rs11743963 | T | C | -0.040 | 0.479 | 297542 | 0.007 | 6.39E-09 | - |
| rs3020408 | C | A | -0.041 | 0.683 | 297542 | 0.007 | 3.23E-08 | - |
| rs1376770 | T | C | -0.047 | 0.206 | 297542 | 0.009 | 4.45E-08 | - |
| rs57268239 | A | C | 0.052 | 0.204 | 297542 | 0.009 | 1.04E-09 | - |
| rs10884051 | G | A | -0.042 | 0.723 | 297542 | 0.008 | 3.85E-08 | - |
| rs606803 | T | A | -0.040 | 0.626 | 297542 | 0.007 | 1.48E-08 | - |
| rs2053606 | C | T | 0.039 | 0.385 | 297542 | 0.007 | 3.63E-08 | - |
| rs2277499 | T | G | -0.041 | 0.330 | 297542 | 0.007 | 2.84E-08 | - |
| rs215896 | C | T | 0.045 | 0.247 | 297542 | 0.008 | 2.21E-08 | - |
| rs7192848 | G | A | 0.046 | 0.469 | 297542 | 0.007 | 2.45E-11 | - |
| rs57852066 | A | G | 0.105 | 0.049 | 297542 | 0.016 | 1.93E-11 | - |
| rs111574702 | T | C | 0.098 | 0.042 | 297542 | 0.017 | 7.89E-09 | - |
| rs6504555 | G | A | -0.053 | 0.760 | 297542 | 0.008 | 4.92E-11 | - |
| rs140005264 | T | G | 0.088 | 0.054 | 297542 | 0.015 | 3.63E-09 | - |
| rs9611466 | T | A | 0.048 | 0.320 | 297542 | 0.007 | 6.39E-11 | - |
| **SNPs associated with confounding factors** | | | | | | | | |
| rs769657 | G | A | -0.047 | 0.334 | 297542 | 0.007 | 2.57E-10 | BMI |
| rs6508210 | C | A | 0.046 | 0.487 | 297542 | 0.007 | 2.15E-11 | BMI |
| rs674094 | C | A | -0.047 | 0.644 | 297542 | 0.007 | 6.87E-11 | Average weekly red wine intake |
| rs707928 | G | A | -0.046 | 0.433 | 297542 | 0.007 | 3.34E-11 | Coeliac disease |
| rs12575544 | A | G | 0.042 | 0.417 | 297542 | 0.007 | 3.18E-09 | Ever smoked |
